# Early-Life Body Mass Index and Inflammatory Bowel Disease Risk: A Scandinavian Birth Cohort Study

**DOI:** 10.1093/ibd/izaf167

**Published:** 2025-08-25

**Authors:** Tereza Lerchova, Johnny Ludvigsson, Staffan Mårild, Henrik Imberg, Björn Andersson, Ketil Størdal, Karl Mårild

**Affiliations:** Department of Pediatrics, Institute of Clinical Sciences, Sahlgrenska Academy, University of Gothenburg, Gothenburg, Sweden; Division of Pediatrics, Department of Biomedical and Clinical Sciences, Linköping University, Linköping, Sweden; Crown Princess Victoria Children’s Hospital, Linköping, Sweden; Department of Pediatrics, Institute of Clinical Sciences, Sahlgrenska Academy, University of Gothenburg, Gothenburg, Sweden; Statistiska Konsultgruppen Sweden, Gothenburg, Sweden; Department of Molecular and Clinical Medicine, Institute of Medicine, Sahlgrenska Academy, University of Gothenburg, Gothenburg, Sweden; Bioinformatics and Data Centre, Sahlgrenska Academy, University of Gothenburg, Gothenburg, Sweden; Department of Pediatrics Research, Faculty of Medicine, University of Oslo, Oslo, Norway; Department of Pediatrics and Adolescent Medicine, Oslo University Hospital, Oslo, Norway; Department of Pediatrics, Institute of Clinical Sciences, Sahlgrenska Academy, University of Gothenburg, Gothenburg, Sweden; Department of Pediatrics, Queen Silvia Children’s Hospital, Gothenburg, Sweden

**Keywords:** obesity, ulcerative colitis, childhood

## Abstract

**Introduction:**

Childhood overweight and obesity are emerging global health issues with potential implications for immune function. We aimed to investigate childhood body mass index (BMI) as a risk factor for later inflammatory bowel disease (IBD).

**Methods:**

ABIS (Sweden) and MoBa (Norway) are population-based cohorts following participants prospectively from birth (1997–2009) until 2023. We retrieved anthropometric data at birth, 1, 3, and 7–8 years to examine the association of age-specific standardized BMI percentiles and categories (underweight, normal [reference), overweight, obesity) with later IBD. We also analyzed IBD risk according to BMI trajectories across ages. IBD diagnosis was identified in national health registries. Cohort-specific hazard ratios (aHRs) were adjusted for sociodemographics, parental BMI, IBD, and smoking and pooled using a random-effects model.

**Results:**

Overall, among 54 890 children with 803 444 person-years of follow-up, we identified 246 IBD events. Eight-year-olds living with obesity had a 5-fold increased risk of ulcerative colitis (pooled aHR = 5.10; 95% CI , 1.51–17.27), but not a significantly increased risk of Crohn’s disease (pooled aHR = 1.38; 95% CI , 0.24–7.98) and IBD overall (pooled aHR = 1.89; 95% CI , 0.71–5.04). Children with overweight or obesity at age 3 had no increased risk of IBD compared to normal-weight children (pooled aHR = 1.15, 95% CI , 0.74–1.77; and 1.05, 95% CI , 0.43–2.58, respectively). Early-life BMI trajectories were not consistently associated with IBD.

**Conclusion:**

In this Scandinavian birth cohort, 7–8 year-old children with obesity had an increased risk of developing ulcerative colitis later in life. Given the high prevalence of childhood obesity, this observation should be corroborated and possible mechanisms behind the association clarified.

Key Messages **What’s already known?**

- Obesity alters immune regulation, promoting inflammation and potentially increasing susceptibility to immune-mediated diseases.
- In adults, obesity has been associated with an increased risk of developing inflammatory bowel disease (IBD).

**What is new here?**

- This binational birth cohort study examines prospectively recorded data on early-life body mass index (BMI) as a risk factor for later IBD.
- Living with obesity at age 7–8 years was associated with a 5-fold increased risk of childhood- and early adult-onset ulcerative colitis but not Crohn’s disease.

**How can this study help patient care?**

- Our observations support the hypothesis that encouraging a healthy lifestyle early in life could play a role in reducing the risk of developing IBD later on.
- As the preschool years are considered important for obesity development, early interventions to support healthy weight—particularly before puberty—might contribute to lowering long-term health risks.

## Introduction

The prevalence of childhood overweight and obesity has surged globally in the last 3 decades, becoming a major public health issue.^1^ While an increased risk of type 2 diabetes, cardiovascular disease, and psychological problems are well documented,^2^ there is concern that living with childhood overweight or obesity may also impact immune function and increase the risk of immune-mediated diseases. Compelling evidence suggests that immune regulation is altered in individuals with obesity, with excess release of pro-inflammatory cytokines prompting chronic low-grade inflammation.^3^ Obesity may also be associated with a shift in the gut microbiome composition, affecting immune function and overall health outcomes.^3^

Children living with obesity have been found to have an increased susceptibility to autoimmune diseases such as rheumatoid arthritis.^4^ While adults with obesity have been shown to have an increased risk of inflammatory bowel disease (IBD),^5^ pediatric studies in this field remain scarce, and obesity in later childhood or adolescence has only inconsistently been linked to subsequent IBD^6^^,^^7^ (Table S1). There is no data on whether IBD risk may be related to growth and BMI development in early childhood, which is a particularly vulnerable period of growth and development with potentially long-term ramifications for health.

In this nationwide Scandinavian birth cohort study, we aimed to examine BMI in early life, particularly overweight or obesity, as a possible risk factor for childhood and early adult-onset IBD.

## Materials and Methods

### Study Population

**Figure 1.**
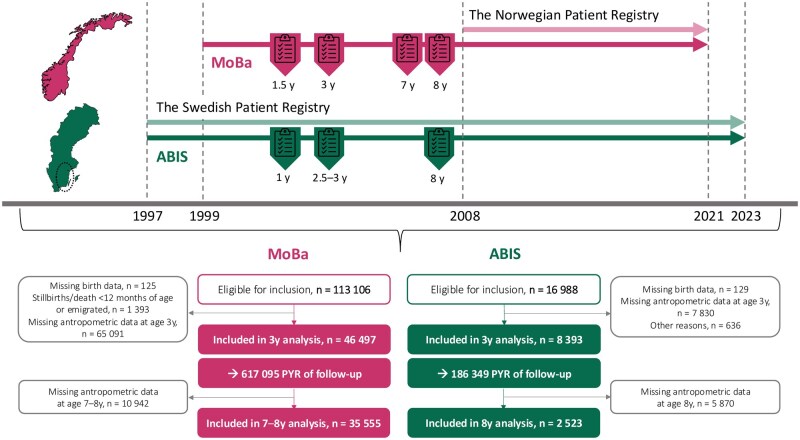
Flow chart of the study design and data collection process for MoBa and ABIS cohort participants. If 8-year data were unavailable, we used growth data reported at 7 years of age; anthropometric data were available for 31 924 children at age 7 years and for 25 959 children at age 8 years. Abbreviations: ABIS, All Babies in Southeast Sweden; MoBa, The Norwegian Mother, Father, and Child Cohort Study; PYR, person-years.

We retrieved prospectively collected data from 2 large-scale population-based birth cohort studies: All Babies in Southeast Sweden (ABIS)^8^ and the Norwegian Mother, Father, and Child Cohort Study (MoBa).^9^ ABIS invited all 21 700 children born in Southeast Sweden from October 1997 to October 1999, out of whom 17 055 consented to participate (participation rate of 79%).^8^ The MoBa cohort invited pregnant women across Norway between July 1999 and December 2008 with data on >114 000 children (participation rate 41%).^9^ This study was restricted to children with available baseline characteristics and anthropometric data at 1 year, 3, and 7–8 years (Figure 1). The personal identity numbers linked ABIS and MoBa participants to high-quality individual-level data from national health registers.^10^^,^^11^

### Exposures

Parents of both cohorts reported their child’s length/height and weight at ages 1, 3, and 7–8 years, which were used to calculate age-specific body mass index (BMI, kg/m^2^). Norwegian parents were instructed to transfer length/height and weight measurements from Child Health Service visits, if available. Regarding the later age category, in MoBa, we primarily used anthropometric data reported at age 8 years, or when such data were missing, we used similar data given at age 7 years; in ABIS, only data from age 8 years were available (Table S2). Childhood BMI categories were defined using age- and sex-specific cut-off points for underweight, normal weight, overweight, and obesity derived from the International Obesity Task Force (IOTF) classification^12^ (Table S2). The IOTF classification is based on data collected from multiple countries and is intended to provide a global reference for assessing overweight and obesity in children and adolescents.

We also examined trajectories of age-specific BMI percentiles (ie, decrease or increase BMI) between birth and 1 year, birth and 3 years, and between 3 and 7–8 years of age. Data on birth weight and length were retrieved from medical birth registries of Norway and Sweden.^13^^,^^14^ Motivated by others,^15^ the age-specific BMI percentile trajectories were based on each cohort’s internal data distribution. These internally standardized BMI percentiles were tested for linear and non-linear associations with the outcome by evaluating hazard ratios (HRs) at selected percentiles (5th, 10th, 25th, 75th, 90th, and 95th) relative to the median BMI level (ie, the 50th percentile).

### Outcome

The diagnosis of IBD and its subtypes was defined by at least 2 International Classification of Diseases (ICD)-10 codes in the National Patient Registries of Sweden and Norway, respectively^10^^,^^11^ (Table S3). Diagnoses were captured throughout 2023 in ABIS and 2021 in MoBa. This registry-based diagnostic algorithm has shown ≥93% positive predictive value for a clinical IBD diagnosis, as validated by medical record reviews.^16^^,^^17^ The time of diagnosis was defined by the first (out of a minimum of 2) recorded ICD-10 codes. Distinct ICD-10 codes for each subtype distinguished UC from CD.^18^ Events classified as IBD-unclassified (IBD-U) were included in the outcome of any IBD but were not separately analyzed due to a higher risk of misclassification.^19^

### Other Data

Based on the current literature, we preselected the following covariates possibly linked to childhood BMI and IBD: child’s birth weight and exclusive breastfeeding duration,^20^ parental IBD^21^ and country of origin, maternal prepregnancy and paternal BMI, maternal education level,^22^ and smoking habits during pregnancy.^23^ This information was retrieved from the at-birth and 1-year questionnaires in ABIS, the early pregnancy and 6-month questionnaires in MoBa, and the Medical Birth Registries of Sweden and Norway^13^^,^^14^ (Table S4). Additionally, we collected questionnaire data on early-life diet quality,^24^ screen time, and physical activity^25^ (Table S4).

### Statistical Analysis

Cox proportional-hazard regression was used to estimate HRs with 95% CIs for IBD, Crohn’s disease (CD), and ulcerative colitis (UC) separately in each cohort. Visual data assessment, examination of Schoenfeld residuals,^26^ and interaction-with-time analyses confirmed the validity of the proportional-hazards assumption. Participants with a diagnosis of UC or IBD-U were ignored in the CD-specific analysis and vice versa in the UC analyses. The follow-up started at ages 1 and 3 years. However, to reduce the risk of reverse causation, we applied a 1-year lag period in the 7 to 8–year analyses, meaning follow-up in this analysis started at 8 or 9 years. Follow-up ended at the time of IBD diagnosis or censoring at the end of data capture (December 31, 2023 [ABIS) and December 31, 2021 [MoBa]). Restricted cubic splines were used to assess relationships between BMI percentiles at ages 3 and 7–8 years, and BMI percentile trajectories at ages 0–1, 0–3, and 3–7 or 8 years and the future risk of IBD and its subtypes.^27^ The analysis of BMI percentile trajectories from ages 3 to 7–8 years focused primarily on IBD overall and UC outcomes due to the limited number of CD events. We used 2 adjustment models. Model 1 accounted for the child’s birth weight, parental IBD and country of origin, paternal BMI, maternal pre-pregnancy BMI, smoking during pregnancy, and education level. Model 2 also accounted for exclusive breastfeeding duration. Sensitivity analyses were further adjusted for early-life diet quality,^24^ screen time and physical activity.^25^ Cohort-specific estimates were pooled using the DerSimonian-Laird random effects meta-analytical method. The Firth penalized likelihood correction was applied in cases of missing or sparse outcome data to ensure accurate risk estimates.

All analyses were performed with the use of SPSS Statistics, version 29.0 (IBM Corp. Armonk, NY, USA), and R software, version 4.3.2 and 4.2.2 (R Core Team, Vienna, Austria), including the survival, survminer, meta, and metafor packages.

### Ethics

The Swedish and Norwegian ethical committees approved this study.

## Results

### Study Population

This study included 54 890 children (ABIS, *n *= 8393 [48.2% girls]; MoBa, *n* = 46 497 [48.8% girls]) with anthropometric data at 3 years of age. During 803 444 person-years of follow-up, 246 participants were diagnosed with IBD. The median age at diagnosis was 19.0 (interquartile range [IQR] = 15.9–22.1) years in ABIS and 13.3 (IQR = 10.6–15.7) years in MoBa. Accounting for differences in ages at the end of follow-up, there were similar incidence rates of IBD across cohorts (Table S5). Three-year-olds who were overweight or obese had higher parental BMI and lower maternal education levels compared to those with a normal BMI, while other characteristics were broadly similar (Table 1). The median BMI at age 3 was 16.4 (IQR = 15.6–17.3) in ABIS and 16.0 (IQR = 15.1–17.0) in MoBa (Table 1).

**Table 1.** Participant characteristics for ABIS and MoBa overall and divided by body mass index (BMI) at age 3 years.

	ABIS					MoBa				
	Overall	Obesity^a^	Overweight^a^	Normal weight^a^	Underweight^a^	Overall	Obesity^a^	Overweight^a^	Normal weight^a^	Underweight^a^
	(*n *= 8393)	(*n *= 239)	(*n* = 1204)	(*n* = 6260)	(*n* = 690)	(*n* = 46 497)	(*n* = 1042)	(*n* = 5050)	(*n* = 32 766)	(*n* = 7639)
BMI at age 3 years										
Median (Q1, Q3)	16.4 (15.6,17.3)	20.2 (19.8,21.0)	18.3 (18.0,18.7)	16.3 (15.6,16.9)	14.3 (13.9,14.5)	16.0 (15.1,17.0)	20.2 (19.8,21.0)	18.3 (18.0,18.7)	16.1 (15.5,16.7)	14.1 (13.6,14.5)
Sex, n (%)										
Female	4043 (48.2)	128 (53.6)	594 (49.3)	2961 (47.3)	360 (52.2)	23 788 (51.2)	536 (51.4)	2463 (48.8)	17 019 (51.9)	3770 (49.4)
Time of follow-up (years)^b^										
Median (Q1, Q3)	22.3 (21.8,22.7)	22.2 (21.8,22.7)	22.2 (21.8,22.7)	22.3 (21.8,22.7)	22.2 (21.8,22.7)	13.2 (11.7,14.8)	13.3 (11.9,14.9)	13.1 (11.7,14.7)	13.2 (11.7,14.9)	13.2 (11.8,14.9)
Exclusive breastfeeding duration, n (%)^c^										
<4.0 months	1921 (22.9)	60 (25.1)	279 (23.2)	1420 (22.7)	162 (23.5)	18 160 (39.1)	435 (41.7)	2057 (40.7)	12 607 (38.5)	3061 (40.1)
4.0–5.9 months	2404 (28.6)	68 (28.5)	324 (26.9)	1800 (28.8)	212 (30.7)	20 664 (44.4)	456 (43.8)	2160 (42.8)	14 743 (45.0)	3305 (43.3)
≥6.0 months	981 (11.7)	20 (8.4)	145 (12.0)	741 (11.8)	75 (10.9)	6294 (13.5)	119 (11.4)	678 (13.4)	4504 (13.7)	993 (13.0)
Missing	3087 (36.8)	91 (38.1)	456 (37.9)	2299 (36.7)	241 (34.9)	1379 (3.0)	32 (3.1)	155 (3.1)	912 (2.8)	280 (3.7)
Birth weight, *n* (%)^c^										
Normal weight	7893 (94.0)	223 (93.3)	1124 (93.4)	5898 (94.2)	648 (93.9)	44 279 (95.2)	977 (93.8)	4822 (95.5)	31 350 (95.7)	7130 (93.3)
High weight	167 (2.0)	11 (4.6)	47 (3.9)	107 (1.7)	2 (0.3)	720 (1.5)	50 (4.8)	157 (3.1)	454 (1.4)	59 (0.8)
Low weight	247 (2.9)	2 (0.8)	22 (1.8)	184 (2.9)	39 (5.7)	1480 (3.2)	14 (1.3)	67 (1.3)	950 (2.9)	449 (5.9)
Missing	86 (1.0)	3 (1.3)	11 (0.9)	71 (1.1)	1 (0.1)	18 (0.0)	1 (0.1)	4 (0.1)	12 (0.0)	1 (0.0)
Parental IBD, *n* (%)^c^										
No	8290 (98.8)	236 (98.7)	1189 (98.8)	6188 (98.8)	677 (98.1)	45 418 (97.7)	1017 (97.6)	4928 (97.6)	32 011 (97.7)	7462 (97.7)
Yes	103 (1.2)	3 (1.3)	15 (1.2)	72 (1.2)	13 (1.9)	1079 (2.3)	25 (2.4)	122 (2.4)	755 (2.3)	177 (2.3)
Parental origin, *n* (%)^c^										
Norwegian/Swedish	7475 (89.1)	212 (88.7)	1077 (89.5)	5583 (89.2)	603 (87.4)	41 029 (88.2)	935 (89.7)	4484 (88.8)	28 938 (88.3)	6672 (87.3)
Other country	731 (8.7)	21 (8.8)	98 (8.1)	534 (8.5)	78 (11.3)	4443 (9.6)	84 (8.1)	470 (9.3)	3112 (9.5)	777 (10.2)
Missing	187 (2.2)	6 (2.5)	29 (2.4)	143 (2.3)	9 (1.3)	1025 (2.2)	23 (2.2)	96 (1.9)	716 (2.2)	190 (2.5)
Paternal BMI, *n* (%)^c^										
Normal weight	3522 (42.0)	58 (24.3)	426 (35.4)	2720 (43.5)	318 (46.1)	20 119 (43.3)	340 (32.6)	1860 (36.8)	14 275 (43.6)	3644 (47.7)
Obesity	369 (4.4)	27 (11.3)	73 (6.1)	247 (3.9)	22 (3.2)	4337 (9.3)	145 (13.9)	668 (13.2)	2905 (8.9)	619 (8.1)
Overweight	2644 (31.5)	92 (38.5)	423 (35.1)	1942 (31.0)	187 (27.1)	20 001 (43.0)	489 (46.9)	2323 (46.0)	14 157 (43.2)	3032 (39.7)
Underweight	24 (0.3%)	1 (0.4)	3 (0.2)	16 (0.3)	4 (0.6)	83 (0.2)	1 (0.1)	4 (0.1)	53 (0.2)	25 (0.3)
Missing	1834 (21.9)	61 (25.5)	279 (23.2)	1335 (21.3)	159 (23.0)	1957 (4.2)	67 (6.4)	195 (3.9)	1376 (4.2)	319 (4.2)
Maternal BMI, *n* (%)^c^										
Normal weight	4968 (59.2)	110 (46.0)	608 (50.5)	3789 (60.5)	461 (66.8)	30 067 (64.7)	545 (52.3)	3021 (59.8)	21 404 (65.3)	5097 (66.7)
Obesity	716 (8.5)	38 (15.9)	148 (12.3)	497 (7.9)	33 (4.8)	4096 (8.8)	165 (15.8)	596 (11.8)	2739 (8.4)	596 (7.8)
Overweight	1957 (23.3)	78 (32.6)	354 (29.4)	1389 (22.2)	136 (19.7)	9829 (21.1)	274 (26.3)	1228 (24.3)	6894 (21.0)	1433 (18.8)
Underweight	166 (2.0)	1 (0.4)	7 (0.6)	139 (2.2)	19 (2.8)	1263 (2.7)	14 (1.3)	88 (1.7)	860 (2.6)	301 (3.9)
Missing	586 (7.0)	12 (5.0)	87 (7.2)	446 (7.1)	41 (5.9)	1242 (2.7)	44 (4.2)	117 (2.3)	869 (2.7)	212 (2.8)
Maternal education, *n* (%)^c^										
Compulsory school	553 (6.6)	22 (9.2)	86 (7.1)	395 (6.3)	50 (7.2)	2371 (5.1)	97 (9.3)	274 (5.4)	1565 (4.8)	435 (5.7)
High school	4511 (53.7)	139 (58.2)	657 (54.6)	3327 (53.1)	388 (56.2)	12 190 (26.2)	287 (27.5)	1324 (26.2)	8446 (25.8)	2133 (27.9)
University/College	3138 (37.4)	71 (29.7)	432 (35.9)	2394 (38.2)	241 (34.9)	31 436 (67.6)	644 (61.8)	3401 (67.3)	22 415 (68.4)	4976 (65.1)
Missing	191 (2.3)	7 (2.9)	29 (2.4)	144 (2.3)	11 (1.6)	500 (1.1)	14 (1.3)	51 (1.0)	340 (1.0)	95 (1.2)
Maternal smoking during pregnancy, *n* (%)^c^										
No	7496 (89.3)	188 (78.7)	1060 (88.0)	5623 (89.8)	625 (90.6)	42 647 (91.7)	902 (86.6)	4610 (91.3)	30 170 (92.1)	6965 (91.2)
Yes	708 (8.4)	45 (18.8)	114 (9.5)	494 (7.9)	55 (8.0)	3330 (7.2)	130 (12.5)	379 (7.5)	2252 (6.9)	569 (7.4)
Missing	189 (2.3)	6 (2.5)	30 (2.5)	143 (2.3)	10 (1.4)	520 (1.1)	10 (1.0)	61 (1.2)	344 (1.0)	105 (1.4)

a BMI categories were defined using International Obesity Task Force classification age- and sex-specific cut-off values (12).

b Follow-up equals time from age 3 years until the end of follow-up (IBD diagnosis or censoring).

c Definitions of all covariates are detailed in Table S4.

Abbreviations: ABIS, All Babies in Southeast Sweden; BMI, body mass index; IBD, inflammatory bowel disease; MoBa, The Norwegian Mother, Father, and Child Cohort Study.

### Early-Life BMI and Later Risk of IBD

**Figure 2.**
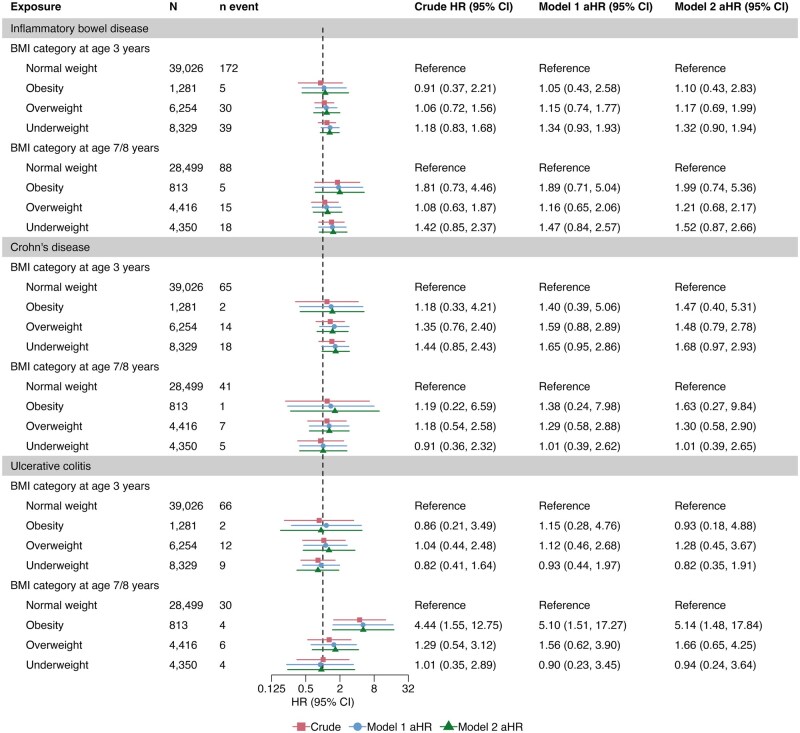
Pooled analyses of the association between childhood body mass index (BMI) and the risk of inflammatory bowel disease, Crohn’s disease, and ulcerative colitis. Adjusted model 1 accounted for the child’s birth weight, parental IBD and country of origin, paternal BMI, maternal pre-pregnancy BMI, smoking status, and education level. Adjusted model 2 accounted for exclusive breastfeeding duration and the covariates included in adjusted model I. Abbreviations: aHR, adjusted hazard ratio; BMI, body mass index; CI, confidence interval; HR, hazard ratio

At age 3 years, 2.3% of children (*n* = 1281 of 54 890) were obese. Adjusting for the child’s birth weight, parental IBD, country of origin, and BMI, maternal smoking and education level, obesity was not associated with subsequent IBD (pooled adjusted [a]HR 1.05, 95% CI, 0.43–2.58) or CD and UC specifically (Figure 2). Neither overweight children, comprising 11.4% of 3-year-olds (*n *= 6254 of 54 890), nor underweight children had a significantly increased risk of later IBD (pooled aHR 1.15, 95% CI, 0.74–1.77; and 1.34, 95% CI, 0.93–1.93, respectively; Figure 2). Also, pooled analyses revealed no statistically significant associations between BMI percentiles at age 3 years and later IBD (Table S10). Cohort-specific estimates for 3-year analyses were consistent with the pooled results (Tables S6–S8).

Children living with obesity at age 7–8 years (2.1%, *n* = 813 of 38 078) were associated with a 5-fold increased risk of developing UC (pooled aHR 5.10, 95% CI, 1.51–17.27) (Figure 2). The corresponding aHRs were 1.89 (95% CI, 0.71–5.05) for IBD and 1.38 (95% CI, 0.24–7.98) for CD. The association with UC remained statistically significant after additional adjustments for exclusive breastfeeding duration (Figure 2) and was similar—though no longer statistically significant—after further adjustment for early-life diet quality, screen time, and physical activity (Table S9). Descriptive analysis showed a normal height distribution among children with UC and obesity, while weight values were skewed toward higher levels (data not shown). Overweight at age 7–8 years (11.6%, *n* = 4416 of 38 078) was not significantly associated with either IBD (pooled aHR 1.16, 95% CI, 0.65–2.06) or its subtypes (Figure 2). Thinness at age 7–8 years was observed in 11.4% of children (*n* = 4350 of 38 078) and yielded a pooled aHR of 1.47 (95% CI, 0.84–2.57) for IBD (Figure 2). Analyses of BMI percentiles at 7–8 years revealed no clear association with later IBD, CD, or UC (Table S11). Cohort-specific analyses were consistent with our pooled analyses (Tables S6–S8).

### BMI Trajectories in Early Childhood and Risk of IBD

We used restricted cubic spline models to examine longitudinal BMI trajectories from 0–1, 0–3, and 3–7 or 8 years (Figures S1–S9). A decelerated BMI trajectory at 0–1 years was associated with an increased risk of UC (model 1), which, however, did not remain after additional adjustment for exclusive breastfeeding duration (Figure S3); similar patterns at 0–1 years were not observed with later CD or IBD risk overall (Figure S1 and S2). BMI development until age 8 was not statistically significantly associated with IBD or its subtypes across all trajectories and adjustment models (Figures S4–S9).

## Discussion

This analysis of 2 population-based birth cohorts, following BMI at ages 1, 3, and 7–8 years, revealed that children living with obesity at 7–8 years had a 5-fold increased risk of subsequent UC, but not IBD or CD development. Overall, BMI at 3 years was not statistically significantly associated with the risk of IBD or its subtypes. Although BMI trajectories in early childhood suggested a possible relationship, they were not consistently associated with IBD risk.

Parallel to the increase in IBD incidence in Western countries, the number of children living with obesity has also grown.^1^^,^^28^ Obesity is frequently associated with chronic low-grade inflammation,^3^ which could play a role in the development of IBD and the progression of the disease course. The adipose tissue, adipocytes, and infiltrating immune cells secrete pro-inflammatory cytokines, such as tumor necrosis factor-alpha and interleukin-6,^3^ that in obese individuals can sustain chronic inflammation and affect gut function.^2^^,^^3^ In obese individuals, endocrinological alterations may also foster an environment conducive to the inflammatory processes of IBD.^2^

While an association between adult obesity and the subsequent risk of IBD,^5^^,^^27^ particularly CD, has been reported in several cohort studies, similar data from children remain scarce. We observed that children living with obesity at age 7–8 years had an increased risk of later UC (aHR 5.10, 95% CI, 1.51–17.27). While the association with UC remained largely unchanged in comprehensively adjusted analyses, it was based on a limited number of children who developed UC, resulting in wide confidence intervals. This finding requires careful interpretation and further validation in prospective studies across diverse cohorts, with longer follow-up and more precise measures of body composition and relevant biomarkers. Nevertheless, if substantiated, these results may have valuable implications, as interventions promoting a healthy early lifestyle would be an attractive target for future IBD prevention strategies.^29^ Preschool years are considered a critical time for the development of obesity and related complications.^30^ Weight loss ameliorates the risks of childhood obesity. However, the risk reduction is affected by the age at which weight loss occurs. In general, evidence suggests that weight loss before puberty is more beneficial,^30^ emphasizing the importance of early preventive strategies.

In 2018, Jensen et al reported findings from a Danish cohort study showing an association between living with obesity at ages 9–13 years and an increased risk of developing CD, but not UC. The authors also found that a 1-unit increase in BMI *z*-score at each age from 7–13 years was associated with a higher risk of CD but a lower risk of UC.^6^ Prenatal factors, nutrition, and socioeconomics often determine growth during the first years of life. Still, as childhood progresses, growth becomes increasingly influenced by hormonal changes, including puberty-related sex steroids, and physical activity.^31^ Also, methodological differences might explain differences between our findings and those of Jensen et al. While our analysis was based on cohorts of children born between 1997 and 2009, Danish authors included children born from 1930–1989, during which childhood obesity was more uncommon and partly linked to other risk factors.^1^ Cohort differences in environment and diagnostic methods available are likely also to have affected the outcome of IBD, which incidence has increased over the past decades.^28^ Additionally, while we focused on IBD development in children and adolescents (median age 19.0 years in ABIS and 13.3  in MoBa), Jensen et al^6^ captured mostly adult diagnoses of IBD (mean age 46.2 years). Risk factors may differ between childhood-onset and adult-onset IBD,^32^ and a substantial fraction of the patients, particularly when initially diagnosed with UC, will change the IBD subtype during the life course.^33^ Finally, unlike in our analysis, Danish authors did not adjust for potential confounding from IBD heredity, parental BMI, lifestyle factors, socioeconomic factors, and smoking.^6^

Our 3-year analysis yielded null results. Early-life BMI often fluctuates considerably, especially during growth spurts and periods of rapid development.^34^ Hence, the BMI trajectory is often more varied in early childhood than in later life (eg, between adolescence and adulthood).^30^ Although at a population level, early-life BMI is linked to later weight status,^35^ it is not a decisive factor, and most toddlers with obesity will not remain obese in adolescence. Instead, BMI tracking seems stronger for obesity at 7–8 years and obesity in adolescence or adulthood.^30^

We also identified a suggestive relationship between BMI trajectories during early childhood and the risk of IBD, particularly UC. While similar trends have been reported in a previous study,^6^ we were unable to consistently confirm these findings throughout all BMI trajectories and adjustment models. Therefore, further prospective research is warranted to explore this potential association.

### Strengths and Limitations

This study’s strengths include using data from 2 population-based Scandinavian birth cohorts, which increases the generalizability of our findings to similar populations while minimizing selection bias. Our prospectively collected data reduces the risk of recall bias and reverse causation. We used well-established international criteria to define categories of BMI,^12^ a measure closely related to adiposity in children across populations,^36^ although its accuracy may vary by age, sex, and ethnicity. Using the unique personal identity number assigned to all citizens in Norway and Sweden, we linked the questionnaire data to national health registries, rendering a virtually complete follow-up for the outcome of IBD.^10^^,^^11^ Our register-based IBD algorithm has a positive predictive value of 93%.^16^^,^^17^ Finally, our dataset allowed us to account for several potential confounders, including early-life diet quality, breastfeeding duration, screen time, physical activity, parental IBD status, BMI, education level, and smoking habits. Comprehensive confounder adjustment is important given the multifactorial nature of childhood obesity, where many risk factors, including diet, have also been linked to IBD risk.^24^ Likewise, given the genetic contributions to childhood IBD and growth,^32^^,^^37^ accounting for IBD heredity and parental BMI is of substantial importance.

As in any longitudinal study, participant attrition limited our sample size, particularly in the analyses of BMI at 7–8 years, with a corresponding influence on statistical power. Although this study included 2 large-scale birth cohorts, the number of children who developed IBD during follow-up was relatively low. This limited case accrual led to wide confidence intervals, especially in the UC analyses, and reduced our ability to detect modest associations, particularly for CD. Therefore, rather than concluding that there is no excess risk of CD or IBD, our findings suggest that strong associations with overweight or obesity at this age are unlikely. Furthermore, because of our use of parent-reported height and weight, we cannot rule out the possibility that erroneous reports may have biased the results towards null and contributed to a type 2 error. However, while the reliability of parent-reported anthropometrics is generally considered high,^38^ given the prospective nature of our data, any misclassification of BMI is unlikely to cause spurious associations with IBD. The observational design limits causal interpretation; therefore, our findings should not be viewed as evidence for specific preventive strategies. We also acknowledge that the study does not assess a cumulative or “hit-on-hit” model of risk, as it was not designed to capture sequential or interacting exposures. Despite adjusting for a wide range of covariates, residual confounding from unmeasured factors should still be considered in the interpretation of our findings. Limitations also include the lack of body composition measures. While BMI remains widely used in epidemiological research, it is still an indirect measure of adiposity and does not distinguish accurately between fat and lean mass, nor does it account for fat distribution. In children, BMI-for-age shows a moderate correlation with actual body fatness, and individuals with similar BMI values can still vary in their metabolic and inflammatory profiles.^39^ Relatively short follow-up limited detection of later-emerging associations between childhood BMI and IBD. The 2 cohorts’ participation rates differed, with MoBa at 41% and ABIS at 79%. Norwegian mothers who smoked during pregnancy or had lower education were less likely to participate in MoBa. Although this self-selection has not been shown to influence the association between exposures and outcomes in previous research,^40^ the generalizability of our findings to subgroups with lower participation rates may be limited.

## Conclusion

In this large-scale binational birth cohort study, obesity at age 7–8 years was associated with an increased risk of developing UC but not CD or IBD overall. No statistically significant association was observed between obesity or overweight in younger children and the risk of IBD.

## Supplementary Material

izaf167_Supplementary_Data

## Data Availability

No additional data are available due to Swedish and Norwegian privacy regulations.
