# Supplementary material for: Early-Life Body Mass Index and Inflammatory Bowel Disease Risk: A Scandinavian Birth Cohort Study
Source: Inflamm Bowel Dis. 2025 Aug 25;31(12):3327–36. doi: 10.1093/ibd/izaf167 (PMC12688070; doi:10.1093/ibd/izaf167)
Supplement: izaf167_Supplementary_Data [file izaf167_supplementary_data.zip › Supplementum_BMI_25October2024_13_REV_004.docx]

**Supplementary materials**

Body Mass Index in Early life and Subsequent Risk of Inflammatory Bowel Disease: A Scandinavian Birth cohort study

Table of Contents

[Supplementary Table S1. Previous research on childhood/adolescent growth and obesity and subsequent risk of inflammatory bowel disease. 3](#_Toc198755238)

[Supplementary Table S2. Harmonization of anthropometric variables between the MoBa and ABIS cohorts. 5](#_Toc198755239)

[Supplementary Table S3. Register-based definition of inflammatory bowel disease. 7](#_Toc198755240)

[Supplementary Table S4. Definitions of covariates for adjustment 8](#_Toc198755241)

[Supplementary Table S5. Number of events, person-years of follow-up, incidence rates and cumulative incidences of IBD and CD, UC in ABIS and MoBa cohorts. 9](#_Toc198755242)

[Supplementary Table S6. Cohort-specific analyses of BMI and later risk of inflammatory bowel disease in ABIS and MoBa. 10](#_Toc198755243)

[Supplementary Table S7. Cohort-specific analyses of BMI and later risk of Crohn’s disease in ABIS and MoBa. 12](#_Toc198755244)

[Supplementary Table S8. Cohort-specific analyses of BMI and later risk of ulcerative colitis in ABIS and MoBa. 14](#_Toc198755245)

[Supplementary Table S9. Sensitivity analysis accounting for early-life diet quality, screen time and physical activity, results of pooled analysis between ABIS and MoBa cohorts. 16](#_Toc198755246)

[Supplementary Table S10. Pooled analyses of restricted cubic spline models of the relationship of BMI percentiles at age 3 years with later IBD, CD, or UC. 17](#_Toc198755247)

[Supplementary Table S11. Pooled analyses of restricted cubic spline models of the relationship of BMI percentiles at age 7–8 years with later IBD, CD, or UC. 18](#_Toc198755248)

[Supplementary Figure S1. Pooled analyses of restricted cubic spline models of the relationship between BMI percentiles trajectories at 0–1 year and inflammatory bowel disease. 19](#_Toc198755249)

[Supplementary Figure S2. Pooled analyses of restricted cubic spline models of the relationship between BMI percentiles trajectories at 0–1 year and Crohn’s disease. 20](#_Toc198755250)

[Supplementary Figure S3. Pooled analyses of restricted cubic spline models of the relationship between BMI percentiles trajectories at 0–1 year and ulcerative colitis. 21](#_Toc198755251)

[Supplementary Figure S4. Pooled analyses of restricted cubic spline models of the relationship between BMI percentiles trajectories at 0–3 years and inflammatory bowel disease. 22](#_Toc198755252)

[Supplementary Figure S5. Pooled analyses of restricted cubic spline models of the relationship between BMI percentiles trajectories at 0–3 years and Crohn’s disease. 23](#_Toc198755253)

[Supplementary Figure S6. Pooled analyses of restricted cubic spline models of the relationship between BMI percentiles trajectories at 0–3 years and ulcerative colitis. 24](#_Toc198755254)

[Supplementary Figure S7. Pooled analyses of restricted cubic spline models of the relationship between BMI percentiles trajectories at 3–7/8 years and inflammatory bowel disease. 25](#_Toc198755255)

[Supplementary Figure S8. Pooled analyses of restricted cubic spline models of the relationship between BMI percentiles trajectories at 3–7/8 years and ulcerative colitis. 26](#_Toc198755256)

[Supplementary Figure S9. Number of participants, events and incidence rates of IBD in the analyses of BMI percentile trajectories at age 0–1 year, 0–3 years, and 3–8 years in the ABIS cohort. 27](#_Toc198755257)

[Supplementary Figure S9. Number of participants, events and incidence rates of IBD in the analyses of BMI percentile trajectories at age 0–1 year, 0–3 years, and 3–7/8 years in the MoBa cohort. 28](#_Toc198755258)

# **Supplementary Table S1.** Previous research on childhood/adolescent growth and obesity and subsequent risk of inflammatory bowel disease.

| First author | Design | Population | Study size | Exposure definition | Result |
| --- | --- | --- | --- | --- | --- |
| Mendall, 2019 [1] | Cohort study | Children  (7–13 years) | 317,030 children | **Height z-scores by age**  Numeric variables at ages 7 – 13 years | **CD:**  **HR** 0.96 (95% CI 0.88–1.04) for birth length  **HR** 1.00 (95% CI 0.85–1.18) for change in height 7-10 years  **HR** 1.02 (95% CI 0.89–1.17) for change in height 10-13 years  **UC:**  **HR** 0.98 (95% CI 0.91–1.05) for birth length  **HR** 0.92 (95% CI 0.81–1.05) for change in height 7-10 years  **HR** 0.95 (95% CI 0.85–1.05) for change in height 10-13 years |
| Jensen, 2018 [2] | Cohort study | Children  (7–13 years) | 316,799 children  (49.4% girls) | **BMI z-scores**  BMI z-scores and changes in BMI z-scores as a numeric and categorical variable (by CDC classification) of underweight, normal weight, overweight, and obesity / loss, no change, and gain | **CD diagnosis before 30 years^1^:**  **HR** 1.2 (95% CI 1.1–1.3) for increasing BMI z-score (per 1 unit increase in BMI z-score) at each age from 7 to 13 years  **HR** 1.40 (95% CI 1.12–1.76) for obesity at age 13 years  **UC:**  **HR** 0.9 (95% CI 0.9–1.0) per 1 unit of BMI z-scores across all ages from 7 to 13 years  **HR** 1.02 (95% CI 0.83–1.24) for obesity at age 13 years |
| Mendall, 2019 [3] | Cohort study | Adolescent  (mean age 19.9 [1.7] years) | 377,957 men | **BMI**  BMI as a categorical variable of underweight, normal weight, overweight, and obesity + numeric variable | **CD:**  **HR** 1.35 (95% CI 1.12–1.62) for underweight,  **HR** 0.83 (95% CI 0.68–1.02) for overweight,  **HR** 1.20 (95% CI 0.75–1.90) for obesity  **UC:**  **HR** 1.03 (95% CI 0.90–1.19) for underweight,  **HR** 0.86 (95% CI 0.75–0.98) for overweight,  **HR** 0.91 (95% CI 0.63–1.30) for obesity |
| Melinder, 2015 [4] | Cohort study | Adolescent  (18–19 years) | 240,984 men | **Height, BMI**  Height grouped into fifths of its distribution; BMI as a categorical variable of underweight (15–18.49), normal weight (18.50–24.99), and overweight/obese (25–60) | **CD:**  **aHR** 1.21 (95% CI 1.01-1.46) for underweight,  **aHR** 0.86 (95% CI 0.66-1.11) for overweight  **UC:**  **aHR** 1.00 (95% CI 0.87-1.16) for underweight,  **aHR** 0.78 (95% CI 0.64-0.95) for overweight |

^1^The authors performed the analyses of CD separately for diagnoses before and after age 30 years due to non-proportional hazards. There was no significant association between childhood BMI and the risk of CD diagnosed after age 30.

aHR: Adjusted hazard ratio, BMI: Body mass index, CD: Crohn’s disease, CDC: Centers for Disease Control and Prevention, HR: Hazard ratio, UC: Ulcerative colitis

# **Supplementary Table S2.** Harmonization of anthropometric variables between the MoBa and ABIS cohorts.

| **Exposure** | **Description** | **MoBa** | **ABIS** |
| --- | --- | --- | --- |
| **Body mass index at 3 years** | MoBa:  1. Number of cm for height and of kg for weight (Approx. 3 years)  ABIS:  Number of kg for weight and of cm for height  BMI categories^1^:  **1) 1^st^ category = thinness**  (≤ 14.83 [boys]; ≤ 14.6 [girls])  **2) 2^nd^ category = normal weight**  (> 14.83 – < 17.85 [boys]; > 14.6 – < 17.64 [girls])  **3) 3^rd^ category = overweight**  (≥ 17.85 – < 19.5 [boys]; ≥ 17.64 – < 19.38 [girls])  **4) 4^th^ category = obesity**  (≥ 19.5 [boys]; ≥ 19.38 [girls]) | 36 months of age (Q1)  What was your child´s height and weight (without clothes) at 3 years? If you know child´s height and weight at 2 years and 15 – 18 months, enter these measurements too. Give the date when the measurements were taken and enter a cross to indicate whether they were taken by you.  (Height in cm / weight in kg / date in DD/MM/YY)  1. Approx. 3 years  2. Approx. 2 years  3. Approx. 15 – 18 mth | 2.5 – 3 years (Q5)  Current weight and length of the child.  (Weight in kg / height in cm) |
| **Body mass index at 7–8 years** | MoBa:  Number of cm for height and of kg for weight  ABIS:  Number of kg for weight and of cm for height  BMI categories^1^:  **1) 1^st^ category = thinness**  (≤ 14.00 [boys]; ≤ 13.83 [girls]); age 7  (≤ 14.13 [boys]; ≤ 14.0 [girls]); age 8  **2) 2^nd^ category = normal weight**  (> 14.00 – < 17.88 [boys]; > 13.83 – < 17.69 [girls]); age 7  (> 14.13 – < 18.41 [boys]; > 14.0 – < 18.28 [girls]); age 8  **3) 3^rd^ category = overweight**  (≥ 17.88 – < 20.59 [boys]; ≥ 17.69 – < 20.39 [girls]), age 7  (≥ 18.41 – < 21.56 [boys]; ≥ 18.28 – < 21.44 [girls]); age 8  **4) 4^th^ category = obesity**  (≥ 20.59 [boys]; ≥ 20.39 [girls]); age 7  (≥ 21.56 [boys]; ≥ 21.44 [girls]); age 8 | 7 years of age (Q1)^2^  What is your child's height and weight now at 7 years of age?  (Height in cm / weight in kg)  8 years of age (Q7)  What is your child's height and weight now at 8 years of age?  (Height in cm / weight in kg) | 8 years of age (Q3)  What is the child's current weight and height?  (Weight in kg / height in cm) |

^1^The categorization was based on age- and sex-specific BMI cut-off values derived from the International Obesity Task Force (IOTF) classification [5].

^2^If 8-year data were unavailable, we used data reported at age 7 years.

ABIS: All Babies in Southeast Sweden, MoBa: The Norwegian Mother, Father and Child Cohort Study, Q: question

# **Supplementary Table S3.** Register-based definition of inflammatory bowel disease.

| **Outcome definitions (≥2 entries of relevant ICD-10 codes for IBD)^1^** | |
| --- | --- |
| **IBD = UC + CD, mixed UC-CD, or K52.3** | IBD diagnosis is defined by ≥ 2 inpatient or non-primary outpatient care visits listing an IBD diagnosis, which has a high positive predictive value in Sweden and Norway [6, 7]. The first recorded diagnosis defined the onset of disease [8]. The most recently recorded ICD-10 code defined the diagnosis for patients shifting between CD and UC, but with only one of the diagnoses during the last five years. Patients with a mix of codes for UC, CD, or indeterminate colitis during follow-up were classified as (“any, non-specific”) IBD [9]. |
| **CD = K50** |  |
| **UC = K51** |  |
| ^1^Definition of IBD were largely consistent with the register-based IBD definition used by Everhov et al. [10]  CD: Crohn's disease, IBD: Inflammatory bowel disease, ICD-10: International Classification of Diseases-10^th^ Revision, UC: Ulcerative colitis | |

# **Supplementary Table S4.** Definitions of covariates for adjustment

|  | **ABIS** | | **MoBa** | |
| --- | --- | --- | --- | --- |
|  | Data source | Definition | Data source | Definition |
| **Birth weight** | MFR | **1)** Low weight = ≤ 2 SD  **2)** Normal weight  **3)** High weight = ≥ 2 SD | MBRN | **1)** Low weight = ≤ 2 SD  **2)** Normal weight  **3)** High weight = ≥ 2 SD |
| **Exclusive breastfeeding duration** | 1-year  questionnaire | **1)** <4.0 months  **2)** 4.0–5.9 months  **3)** ≥6.0 months | 6-months questionnaire | **1)** <4.0 months  **2)** 4.0–5.9 months  **3)** ≥6.0 months |
| **Parental IBD** | At-birth  questionnaire | **1)** No  **2)** Yes | Norwegian Patient Registry | **1)** No  **2)** Yes |
| **Parental country of origin** | At-birth  questionnaire | **1)** Sweden = Sweden has been reported as the country of birth of the mother or father  **2)** Other = Sweden has not been reported as the country of birth of the mother or father | Pregnancy questionnaire | **1)** Norway = Norwegian native speakers (mothers)  **2)** Other = native speakers of other languages (mothers) |
| **Paternal^1^ BMI** | 1-year  questionnaire | **1)** Underweight = BMI <18.5  **2)** Normal weight  = BMI 18.5 – >25  **3)** Overweight = BMI 25 – >30  **4)** Obesity = BMI ≥ 30 | Pregnancy questionnaire | **1)** Underweight = BMI <18.5  **2)** Normal weight  = BMI 18.5 – >25  **3)** Overweight = BMI 25 – >30  **4)** Obesity = BMI ≥ 30 |
| **Maternal^1^ BMI** | MFR | **1)** Underweight = BMI <18.5  **2)** Normal weight  = BMI 18.5 – >25  **3)** Overweight = BMI 25 – >30  **4)** Obesity = BMI ≥ 30 | Pregnancy questionnaire | **1)** Underweight = BMI <18.5  **2)** Normal weight  = BMI 18.5 – >25  **3)** Overweight = BMI 25 – >30  **4)** Obesity = BMI ≥ 30 |
| **Maternal education^2^** | At-birth  questionnaire | **1)** Compulsory school = 9 – 11 years of education  **2)** High school = 12 years of education  **3)** University/College ≥13 years of education | Pregnancy questionnaire | **1)** Compulsory school = 9 – 11 years of education  **2)** High school = 12 years of education  **3)** University/College ≥13 years of education |
| **Maternal smoking during pregnancy^2^** | At-birth  questionnaire | **1)** No  **2)** Yes | Pregnancy and at 6-months questionnaire | **1)** No  **2)** Yes |
| **Physical activity at age 3^2^** | 3-year  questionnaire | **1)** High = ≥2 hours/day  **2)** Low = <2 hours/day | 3-year  questionnaire | **1)** High = ≥2 hours/day  **2)** Low = <2 hours/day |
| **Screen time at age 3^2^** | 3-year  questionnaire | **1)** High = ≥1 hour/day  **2)** Low = < 1hour/day | 3-year  questionnaire | **1)** High = ≥1 hour/day  **2)** Low = < 1hour/day |
| **Diet quality at age 1^2^** | 1-year  questionnaire | **1)** High  **2)** Medium  **3)** Low | 1-year  questionnaire | **1)** High  **2)** Medium  **3)** Low |

^1^The categorization of parental BMI was based on World Health Organization criteria [11].

^2^Maternal education, smoking habits, child’s physical activity, screen time and diet quality are detailed in previous studies [12-15].

ABIS: All Babies in Southeast Sweden, BMI: Body mass index, IBD: Inflammatory bowel disease, MBRN: Medical Birth Registry of Norway, MFR: Medical Birth Registry of Sweden (In Swedish, Medicinska Födelseregistret), MoBa: The Norwegian Mother, Father, and Child Cohort Study, SD: Standard deviation

# **Supplementary Table S5.** Number of events, person-years of follow-up, incidence rates and cumulative incidences of IBD and CD, UC in ABIS and MoBa cohorts.

|  | N event | PYR of follow-up | Incidence rate per 100,000 PYR (95%CI) | Cumulative incidence (%) at the end of  follow-up^1^ | Cumulative incidence (%) by <16 years of age^2^ |
| --- | --- | --- | --- | --- | --- |
| **ABIS** |  |  |  |  |  |
| IBD^3^ | 81 | 186,349 | 43.47 (34.52, 54.03) | 0.97 (0.77, 1.20) | 0.25 (0.15, 0.38) |
| CD | 26 | 186,692 | 13.93 (9.10, 20.41) | 0.31 (0.20, 0.45) | 0.13 (0.07, 0.23) |
| UC | 46 | 186,612 | 24.56 (18.05, 32.88) | 0.55 (0.40, 0.73) | 0.10 (0.04, 0.19) |
| **MoBa** |  |  |  |  |  |
| IBD^3^ | 165 | 617,095 | 26.74 (22.81, 31.14) | 0.35 (0.30, 0.41) | 0.29 (0.24, 0.34) |
| CD | 73 | 617,498 | 11.82 (9.27, 14.86) | 0.16 (0.12, 0.20) | 0.13 (0.10, 0.17) |
| UC | 43 | 617,596 | 6.96 (5.04, 9.38) | 0.09 (0.07, 0.13) | 0.06 (0.08, 0.14) |

^1^End of follow-up was December 31, 2023 (ABIS) and December 31, 2021 (MoBa).

^2^Due to the different follow-up times in ABIS and MoBa, we restricted IBD events to those diagnosed before 16

years of age.

^3^Diagnisis of IBD includes CD, UC and IBD-U events.

ABIS: All Babies in Southeast Sweden study, CD: Crohn’s disease, CI: Confidence interval, IBD: Inflammatory bowel disease, IBD-U: IBD-unclassified, MoBa: The Norwegian Mother, Father and Child Cohort Study, PYR: Person-year, UC: Ulcerative colitis.

# **Supplementary Table S6.** Cohort-specific analyses of BMI and later risk of inflammatory bowel disease in ABIS and MoBa.

| Characteristic | Incidence per 100'000 PY (95% CI) | N | | Event N | | Crude model  (HR 95% CI) | | Adjusted model I^1^  (aHR 95% CI) | | Adjusted model II^2^  (aHR 95% CI) | |  |
| --- | --- | --- | --- | --- | --- | --- | --- | --- | --- | --- | --- | --- |
| BMI categories at age 3 years^3^ | |  | |  | |  |  |  |  |  |  |  |
| ABIS |  |  | |  | |  |  |  |  |  |  |  |
| *Normal weight (reference)* | 42.4(32.3,54.7) | 6,260 | | 59 | | — | — | — | — | — | — |  |
| *Obesity* | 18.9(0.5,105.1) | 239 | | 1 | | 0,44 | (0.06 - 3.21) | 0,71 | (0.10 - 5.25) |  | — ^4^ |  |
| *Overweight* | 52.4(28.7,88) | 1,204 | | 14 | | 1,24 | (0.69 - 2.21) | 1,51 | (0.76 - 2.97) | 1,59 | (0.74 - 3.41) |  |
| *Underweight* | 45.7(18.4,94.1) | 690 | | 7 | | 1,08 | (0.49 - 2.36) | 1,19 | (0.47 - 3.05) | 1,05 | (0.37 - 3.01) |  |
| MoBa |  |  | |  | |  |  |  |  |  |  |  |
| *Normal weight (reference)* | 26(21.4,31.2) | 32,766 | | 113 | | — | — | — | — | — | — |  |
| *Obesity* | 28.7(7.8,73.4) | 1,042 | | 4 | | 1,09 | (0.40 - 2.96) | 1,16 | (0.43 - 3.16) | 1,22 | (0.45 - 3.33) |  |
| *Overweight* | 24(13.7,39) | 5,050 | | 16 | | 0,93 | (0.55 - 1.57) | 0,96 | (0.55 - 1.65) | 0,94 | (0.54 - 1.66) |  |
| *Underweight* | 31.5(21.5,44.5) | 7,639 | | 32 | | 1,21 | (0.82 - 1.79) | 1,37 | (0.92 - 2.04) | 1,35 | (0.90 - 2.05) |  |
| BMI categories at age 7-8 years^3^ | | |  | |  |  |  |  |  |  |  | |
| ABIS |  | |  | |  |  |  |  |  |  |  | |
| *Normal weight (reference)* | 66.7(41.3,101.9) | | 1,936 | | 21 | — | — | — | — | — | — | |
| *Obesity* | 94.7(2.4,527.9) | | 65 | | 1 | 1,43 | (0.19 - 10.65) |  | — ^4^ |  | — ^4^ | |
| *Overweight* | 61.6(12.7,180) | | 298 | | 3 | 0,92 | (0.28 - 3.10) | 0,79 | (0.18 - 3.56) | 0,93 | (0.20 - 4.33) | |
| *Underweight* | 55.1(6.7,198.9) | | 224 | | 2 | 0,82 | (0.19 - 3.51) | 0,51 | (0.07 - 4.00) | 0,56 | (0.07 - 4.49) | |
| MoBa |  | |  | |  |  |  |  |  |  |  | |
| *Normal weight (reference)* | 33.9(26.3,43.1) | | 26,563 | | 67 | — | — | — | — | — | — | |
| *Obesity* | 67.7(18.5,173.4) | | 748 | | 4 | 1,92 | (0.70 - 5.27) | 2,11 | (0.75 - 5.98) | 2,17 | (0.76 - 6.15) | |
| *Overweight* | 38.6(19.9,67.4) | | 4,118 | | 12 | 1,13 | (0.61 - 2.08) | 1,20 | (0.64 - 2.25) | 1,23 | (0.65 - 2.31) | |
| *Underweight* | 52.1(29.8,84.6) | | 4,126 | | 16 | 1,54 | (0.89 - 2.65) | 1,58 | (0.88 - 2.84) | 1,61 | (0.89 - 2.90) | |

^1^Adjusted Model I accounted for the child’s birth weight, parental IBD and country of origin, paternal BMI, maternal pre-pregnancy BMI, smoking during pregnancy and education level. All covariates are detailed in Supplementary Table S3.

^2^Adjusted Model II accounted for exclusive breastfeeding duration and the covariates included in Model I.

^3^The BMI categories were based on age- and sex-specific BMI cut-off values derived from IOTF classification [5].

^4^Model did not converge due to a limited number of events; further analysis was conducted using estimates obtained with Penalized Firth correction.

ABIS: All Babies in Southeast Sweden, aHR: Adjusted hazard ratio, BMI: Body mass index, CI: Confidence interval, HR: Hazard ratio, IOTF: International Obesity Task Force classification, MoBa: The Norwegian Mother, Father, and Child Cohort Study, N: Number, PY: Person-years, y: Year

# **Supplementary Table S7.** Cohort-specific analyses of BMI and later risk of Crohn’s disease in ABIS and MoBa.

| Characteristic | Incidence per 100'000 PY (95% CI) | N | Event N | Crude model  (HR 95% CI) | | Adjusted model I^1^  (HR 95% CI) | | Adjusted model II^2^  (HR 95% CI) | |
| --- | --- | --- | --- | --- | --- | --- | --- | --- | --- |
| BMI categories at age 3 years^3^ | |  |  |  |  |  |  |  |  |
| ABIS |  |  |  |  |  |  |  |  |  |
| *Normal weight (reference)* | 13.6(8.2,21.3) | 6,260 | 19 | — | — | — | — | — | — |
| *Obesity* | 0(0,69.4) | 239 | 0 |  | — ^4^ |  | — ^4^ |  | — ^4^ |
| *Overweight* | 18.7(6.1,43.6) | 1,204 | 5 | 1,37 | (0.51 - 3.67) | 1,89 | (0.67 - 5.30) | 1,37 | (0.38 - 4.85) |
| *Underweight* | 13(1.6,47.1) | 690 | 2 | 0,95 | (0.22 - 4.10) | 0,64 | (0.08 - 4.90) | 0,63 | (0.08 - 4.87) |
| MoBa |  |  |  |  |  |  |  |  |  |
| *Normal weight (reference)* | 10.6(7.7,14.1) | 32,766 | 46 | — | — | — | — | — | — |
| *Obesity* | 14.3(1.7,51.7) | 1,042 | 2 | 1,35 | (0.33 - 5.55) | 1,54 | (0.37 - 6.39) | 1,58 | (0.38 - 6.55) |
| *Overweight* | 13.5(6.2,25.6) | 5,050 | 9 | 1,28 | (0.63 - 2.63) | 1,44 | (0.70 - 2.96) | 1,47 | (0.71 - 3.04) |
| *Underweight* | 15.7(9,25.6) | 7,639 | 16 | 1,49 | (0.84 - 2.63) | 1,75 | (0.98 - 3.12) | 1,80 | (1.00 - 3.21) |
| BMI categories at age 7-8 years^3^ | |  |  |  |  |  |  |  |  |
| ABIS |  |  |  |  |  |  |  |  |  |
| *Normal weight (reference)* | 15.8(5.1,36.9) | 1,936 | 5 | — | — | — | — | — | — |
| *Obesity* | 0(0,348.2) | 65 | 0 |  | — ^4^ |  | — ^4^ |  | — ^4^ |
| *Overweight* | 0(0,75.6) | 298 | 0 |  | — ^4^ |  | — ^4^ |  | — ^4^ |
| *Underweight* | 27.5(0.7,153.3) | 224 | 1 | 1,73 | (0.20 -14.82) | 2,14 | (0.24 - 19.48) | 2,48 | (0.25 - 24.26) |
| MoBa |  |  |  |  |  |  |  |  |  |
| *Normal weight (reference)* | 18.2(12.8,25.2) | 26,563 | 36 | — | — | — | — | — | — |
| *Obesity* | 16.9(0.4,94.2) | 748 | 1 | 0,89 | (0.12 - 6.50) | 1,10 | (0.15 - 8.22) | 1,11 | (0.15 - 8.34) |
| *Overweight* | 22.5(9.1,46.4) | 4,118 | 7 | 1,23 | (0.55 - 2.75) | 1,33 | (0.58 - 3.03) | 1,31 | (0.57 - 3.00) |
| *Underweight* | 13(3.5,33.3) | 4,126 | 4 | 0,72 | (0.25 - 2.01) | 0,84 | (0.30 - 2.38) | 0,83 | (0.29 - 2.36) |

^1^Adjusted Model I accounted for the child’s birth weight, parental IBD and country of origin, paternal BMI, maternal pre-pregnancy BMI, smoking during pregnancy and education level. All covariates are detailed in Supplementary Table S3.

^2^Adjusted Model II accounted for exclusive breastfeeding duration and the covariates included in Model I.

^3^The BMI categories were based on age- and sex-specific BMI cut-off values derived from IOTF classification [5].

^4^Model did not converge due to a limited number of events; further analysis was conducted using estimates obtained with Penalized Firth correction.

ABIS: All Babies in Southeast Sweden, aHR: Adjusted hazard ratio, BMI: Body mass index, CI: Confidence interval, HR: Hazard ratio, IOTF: International Obesity Task Force classification, MoBa: The Norwegian Mother, Father, and Child Cohort Study, N: Number, PY: Person-years, y: Year

# **Supplementary Table S8.** Cohort-specific analyses of BMI and later risk of ulcerative colitis in ABIS and MoBa.

| Characteristic | Incidence per 100'000 PY (95% CI) | N | Event N | Crude model  (HR 95% CI) | | Adjusted model I^1^  (HR 95% CI) | | Adjusted model II^2^  (HR 95% CI) | |
| --- | --- | --- | --- | --- | --- | --- | --- | --- | --- |
| BMI categories at age 3 years^3^ | |  |  |  |  |  |  |  |  |
| ABIS |  |  |  |  |  |  |  |  |  |
| *Normal weight (reference)* | 23(15.7,32.4) | 6,260 | 32 | — | — | — | — | — | — |
| *Obesity* | 18.9(0.5,105.1) | 239 | 1 | 0,82 | (0.11 - 6.00) | 1,51 | (0.20 - 11.46) |  | — ^4^ |
| *Overweight* | 33.6(15.4,63.8) | 1,204 | 9 | 1,46 | (0.70 - 3.06) | 1,62 | (0.64 - 4.12) | 2,01 | (0.74 - 5.49) |
| *Underweight* | 26.1(7.1,66.8) | 690 | 4 | 1,13 | (0.40 - 3.21) | 1,44 | (0.42 - 4.89) | 1,14 | (0.26 - 5.10) |
| MoBa |  |  |  |  |  |  |  |  |  |
| *Normal weight (reference)* | 7.8(5.4,10.9) | 32,766 | 34 | — | — | — | — | — | — |
| *Obesity* | 7.2(0.2,39.9) | 1,042 | 1 | 0,89 | (0.12 - 6.51) | 0,87 | (0.12 - 6.48) | 0,92 | (0.12 - 6.88) |
| *Overweight* | 4.5(0.9,13.1) | 5,050 | 3 | 0,58 | (0.18 - 1.90) | 0,65 | (0.20 - 2.16) | 0,71 | (0.22 - 2.37) |
| *Underweight* | 4.9(1.6,11.5) | 7,639 | 5 | 0,62 | (0.24 - 1.59) | 0,71 | (0.27 - 1.86) | 0,62 | (0.21 - 1.78) |
| BMI categories at age 7-8 years^3^ | |  |  |  |  |  |  |  |  |
| ABIS |  |  |  |  |  |  |  |  |  |
| *Normal weight (reference)* | 44.4(24.3,74.5) | 1,936 | 14 | — | — | — | — | — | — |
| *Obesity* | 94.7(2.4,527.9) | 65 | 1 | 2,15 | (0.28 – 16.39) |  | — ^4^ |  | — ^4^ |
| *Overweight* | 41.1(5,148.3) | 298 | 2 | 0,93 | (0.21 - 4.07) | 1,16 | (0.24 - 5.53) | 1,42 | (0.29 - 6.84) |
| *Underweight* | 27.5(0.7,153.1) | 224 | 1 | 0,62 | (0.08 – 4.69) |  | — ^4^ |  | — ^4^ |
| MoBa |  |  |  |  |  |  |  |  |  |
| *Normal weight (reference)* | 8.1(4.6,13.2) | 26,563 | 16 | — | — | — | — | — | — |
| *Obesity* | 50.8(10.5,148.4) | 748 | 3 | 5,80 | (1.69 - 19.93) | 6,65 | (1.76 - 25.13) | 6,45 | (1.70 - 24.43) |
| *Overweight* | 12.9(3.5,32.9) | 4,118 | 4 | 1,55 | (0.52 - 4.65) | 1,70 | (0.54 - 5.33) | 1,68 | (0.54 - 5.29) |
| *Underweight* | 9.8(2,28.5) | 4,126 | 3 | 1,21 | (0.35 - 4.14) | 1,06 | (0.24 - 4.72) | 1,06 | (0.24 - 4.73) |

^1^Adjusted Model I accounted for the child’s birth weight, parental IBD and country of origin, paternal BMI, maternal pre-pregnancy BMI, smoking during pregnancy and education level. All covariates are detailed in Supplementary Table S3.

^2^Adjusted Model II accounted for exclusive breastfeeding duration and the covariates included in Model I.

^3^The BMI categories were based on age- and sex-specific BMI cut-off values derived from IOTF classification [5].

^4^Model did not converge due to a limited number of events; further analysis was conducted using estimates obtained with Penalized Firth correction.

ABIS: All Babies in Southeast Sweden, aHR: Adjusted hazard ratio, BMI: Body mass index, CI: Confidence interval, HR: Hazard ratio, IOTF: International Obesity Task Force classification, MoBa: The Norwegian Mother, Father, and Child Cohort Study, N: Number, PY: Person-years, y: Year

# **Supplementary Table S9.** Sensitivity analysis accounting for early-life diet quality, screen time and physical activity, results of pooled analysis between ABIS and MoBa cohorts.

|  |  |  | Pooled adjusted HR (95% CI)^1^ | | |
| --- | --- | --- | --- | --- | --- |
|  | **N** | **IBD Event N** | **IBD** | **CD** | **UC** |
| 3-year analysis |  |  |  |  |  |
| BMI categories^2^ |  |  |  |  |  |
| Normal weight | 33,778 | 135 | Reference | Reference | Reference |
| Obesity | 1,051 | 4 | 0.85 (0.31–2.32) | 0.82 (0.16–4.25) | 1.26 (0.30–5.24) |
| Overweight | 5,366 | 25 | 1.17 (0.68–2.01) | 1.52 (0.82–2.83) | 1.15 (0.47–2.82) |
| Underweight | 7,303 | 36 | 1.33 (0.92–1.93) | 1.67 (0.96–2.90) | 0.96 (0.45–2.05) |
| 7-8-year analysis |  |  |  |  |  |
| BMI categories^2^ |  |  |  |  |  |
| Normal weight | 26,151 | 74 | Reference | Reference | Reference |
| Obesity | 741 | 4 | 1.03 (0.32–3.36) | 1.26 (0.21–7.72) | 4.52 (0.79–25.79) |
| Overweight | 4,042 | 13 | 1.14 (0.63–2.07) | 1.30 (0.58–2.93) | 1.46 (0.55–3.92) |
| Underweight | 3,998 | 15 | 1.55 (0.88–2.72) | 1.10 (0.42–2.89) | 0.96 (0.25–3.70) |

^1^Adjustement considers diet quality, screen time and physical activity and the covariates included in Model I (child’s birth weight, parental IBD and country of origin, paternal and maternal pre-pregnancy BMI, smoking during pregnancy and education level); all covariates are detailed in Supplementary Table S3.

^2^The BMI categories were based on age- and sex-specific BMI cut-off values derived from IOTF classification [5].

BMI: Body mass index, CD: Crohn’s disease, CI: Confidence interval, HR: Hazard ratio, IBD: Inflammatory bowel disease, IOTF: International Obesity Task Force classification, UC: Ulcerative colitis

# **Supplementary Table S10.** Pooled analyses of restricted cubic spline models of the relationship of BMI percentiles at age 3 years with later IBD, CD, or UC.

| Exposure | HR (95% CI) | aHR (95% CI), Model I | aHR (95% CI), Model II |
| --- | --- | --- | --- |
| IBD |  |  |  |
| 5th percentile | 1.25 (0.84–1.86) | 1.44 (0.93–2.23) | 1.42 (0.90–2.23) |
| 10th percentile | 1.23 (0.89–1.70) | 1.38 (0.97–1.97) | 1.37 (0.95–1.98) |
| 25th percentile | 1.15 (0.93–1.43) | 1.22 (0.96–1.55) | 1.22 (0.95–1.57) |
| 50th percentile | Reference | Reference | Reference |
| 75th percentile | 0.93 (0.75–1.16) | 1.00 (0.79–1.27) | 0.98 (0.76–1.26) |
| 90th percentile | 0.98 (0.70–1.39) | 1.15 (0.80–1.65) | 1.12 (0.76–1.65) |
| 95th percentile | 1.00 (0.65–1.55) | 1.22 (0.77–1.92) | 1.18 (0.73–1.92) |
| CD |  |  |  |
| 5th percentile | 1.46 (0.78–2.72) | 1.78 (0.92–3.44) | 1.79 (0.92–3.49) |
| 10th percentile | 1.36 (0.82–2.24) | 1.59 (0.93–2.71) | 1.58 (0.92–2.70) |
| 25th percentile | 1.11 (0.79–1.56) | 1.18 (0.82–1.69) | 1.13 (0.78–1.63) |
| 50th percentile | Reference | Reference | Reference |
| 75th percentile | 1.10 (0.77–1.56) | 1.22 (0.84–1.77) | 1.25 (0.85–1.83) |
| 90th percentile | 1.21 (0.72–2.05) | 1.46 (0.84–2.52) | 1.42 (0.80–2.52) |
| 95th percentile | 1.25 (0.65–2.40) | 1.55 (0.79–3.05) | 1.48 (0.73–3.00) |
| UC |  |  |  |
| 5th percentile | 0.95 (0.47–1.90) | 1.02 (0.45–2.30) | 0.92 (0.37–2.24) |
| 10th percentile | 0.94 (0.54–1.65) | 1.01 (0.52–1.95) | 0.93 (0.45–1.90) |
| 25th percentile | 0.93 (0.65–1.33) | 0.99 (0.66–1.50) | 0.97 (0.62–1.51) |
| 50th percentile | Reference | Reference | Reference |
| 75th percentile | 0.97 (0.68–1.40) | 1.02 (0.67–1.56) | 1.00 (0.63–1.58) |
| 90th percentile | 0.86 (0.48–1.54) | 1.07 (0.57–2.02) | 1.01 (0.51–2.01) |
| 95th percentile | 0.82 (0.40–1.70) | 1.09 (0.49–2.40) | 1.01 (0.43–2.38) |

Adjusted Model I accounted for the child’s birth weight, parental IBD and country of origin, paternal BMI status, maternal pre-pregnancy BMI, smoking and education level.

Adjusted Model II accounted for exclusive breastfeeding duration and the covariates included in Model I.

aHR: Adjusted hazard ratio, BMI: Body mass index, CD: Crohn’s disease, CI: Confidence interval, HR: Hazard ratio, IBD: Inflammatory bowel disease, UC: Ulcerative colitis

# **Supplementary Table S11.** Pooled analyses of restricted cubic spline models of the relationship of BMI percentiles at age 7–8 years with later IBD, CD, or UC.

| Exposure | HR (95% CI) | aHR (95% CI), Model I | aHR (95% CI), Model II |
| --- | --- | --- | --- |
| IBD |  |  |  |
| 5th percentile | 1.74 (1.00–3.03) | 1.83 (1.00–3.33) | 2.02 (1.10–3.69) |
| 10th percentile | 1.55 (0.97–2.49) | 1.62 (0.95–2.75) | 1.76 (1.08–2.86) |
| 25th percentile | 1.19 (0.88–1.61) | 1.23 (0.89–1.71) | 1.23 (0.88–1.72) |
| 50th percentile | Reference | Reference | Reference |
| 75th percentile | 1.17 (0.85–1.60) | 1.13 (0.80–1.59) | 1.23 (0.87–1.76) |
| 90th percentile | 1.37 (0.76–2.46) | 1.25 (0.57–2.74) | 1.52 (0.91–2.53) |
| 95th percentile | 1.47 (0.74–2.93) | 1.29 (0.47–3.57) | 1.64 (0.87–3.07) |
| CD |  |  |  |
| 5th percentile | 1.30 (0.53–3.20) | 1.52 (0.60–3.84) | 1.61 (0.63–4.13) |
| 10th percentile | 1.13 (0.55–2.31) | 1.26 (0.60–2.65) | 1.35 (0.64–2.85) |
| 25th percentile | 0.84 (0.53–1.32) | 0.83 (0.38–1.80) | 0.94 (0.58–1.52) |
| 50th percentile | Reference | Reference | Reference |
| 75th percentile | 1.44 (0.86–2.41) | 1.35 (0.80–2.30) | 1.39 (0.82–2.37) |
| 90th percentile | 1.28 (0.25–6.40) | 1.51 (0.72–3.17) | 1.53 (0.73–3.23) |
| 95th percentile | 1.19 (0.15–9.52) | 1.53 (0.53–4.38) | 1.58 (0.64–3.93) |
| UC |  |  |  |
| 5th percentile | 1.33 (0.40–4.45) | 1.25 (0.25–6.22) | 1.58 (0.43–5.76) |
| 10th percentile | 1.33 (0.49–3.64) | 1.31 (0.35–4.89) | 1.56 (0.55–4.43) |
| 25th percentile | 1.32 (0.77–2.26) | 1.47 (0.77–2.79) | 1.42 (0.74–2.72) |
| 50th percentile | Reference | Reference | Reference |
| 75th percentile | 1.03 (0.61–1.74) | 0.92 (0.49–1.71) | 1.08 (0.56–2.07) |
| 90th percentile | 1.67 (0.61–4.60) | 1.65 (0.56–4.86) | 1.98 (0.85–4.61) |
| 95th percentile | 2.03 (0.59–6.98) | 2.13 (0.59–7.68) | 2.52 (0.90–7.05) |

Adjusted Model I accounted for the child’s birth weight, parental IBD and country of origin, paternal BMI status, maternal pre-pregnancy BMI, smoking and education level.

Adjusted Model II accounted for exclusive breastfeeding duration and the covariates included in Model I.

aHR: Adjusted hazard ratio, BMI: Body mass index, CD: Crohn’s disease, CI: Confidence interval, HR: Hazard ratio, IBD: Inflammatory bowel disease, UC: Ulcerative colitis

# **Supplementary Figure S1**. Pooled analyses of restricted cubic spline models of the relationship between BMI percentiles trajectories at 0–1 year and inflammatory bowel disease.


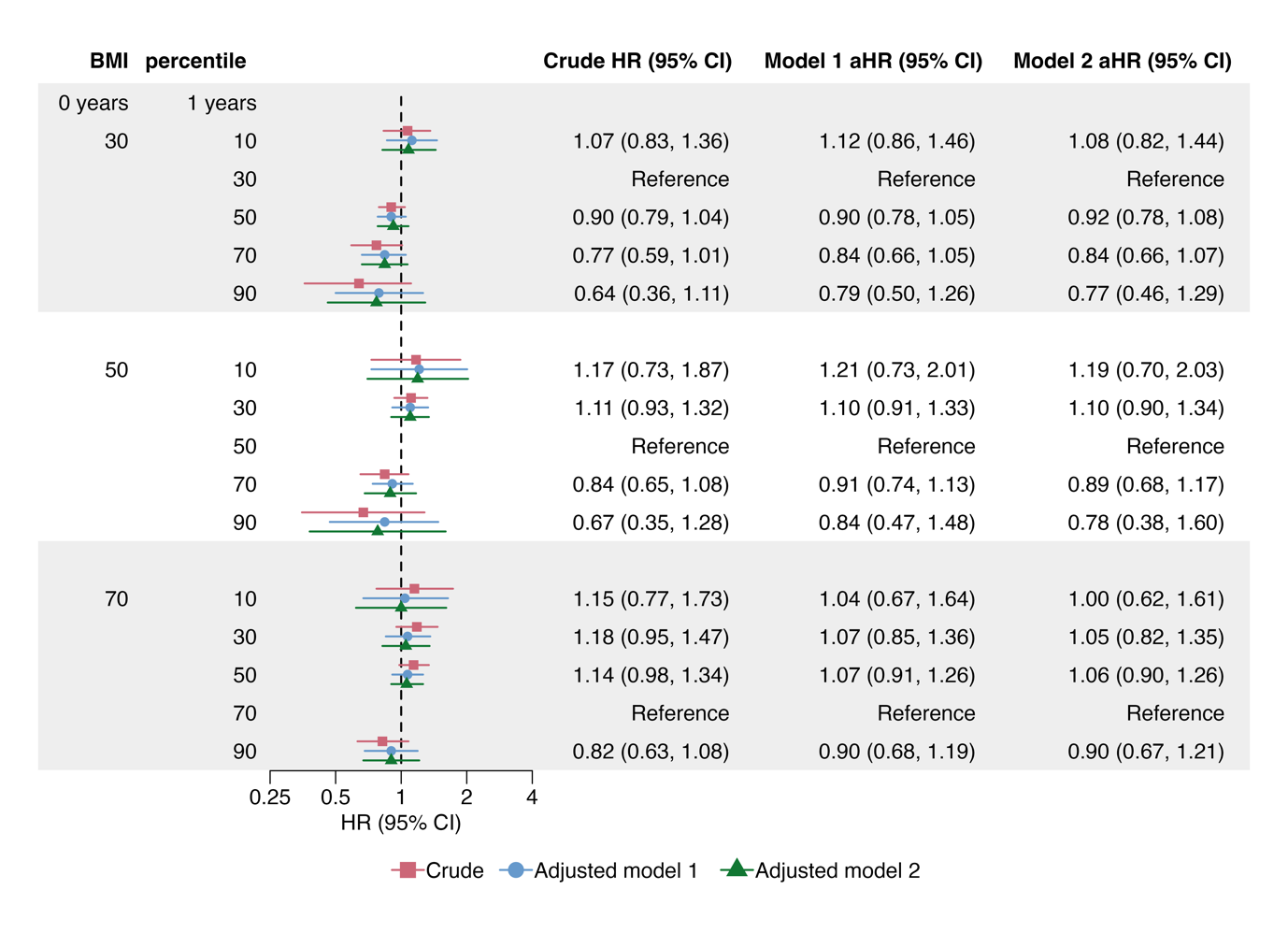


Analyses of BMI trajectories in children aged 0–1 years followed changes from the 30^th^, 50^th^, and 70^th^ BMI percentiles to the 10^th^, 30^th^, 50^th^, 70^th^, and 90^th^ percentiles, considering children who remained at the same percentile as the reference group. The number of participants in each analysis is detailed in Supplementary Figures S14 and S15.

Adjusted Model I accounted for the child’s birth weight, parental IBD and country of origin, paternal BMI status, maternal pre-pregnancy BMI, smoking and education level.

Adjusted Model II accounted for exclusive breastfeeding duration and the covariates included in Model I.

aHR: Adjusted hazard ratio, BMI: Body mass index, CI: Confidence interval, HR: Hazard ratio

# **Supplementary Figure S2.** Pooled analyses of restricted cubic spline models of the relationship between BMI percentiles trajectories at 0–1 year and Crohn’s disease.


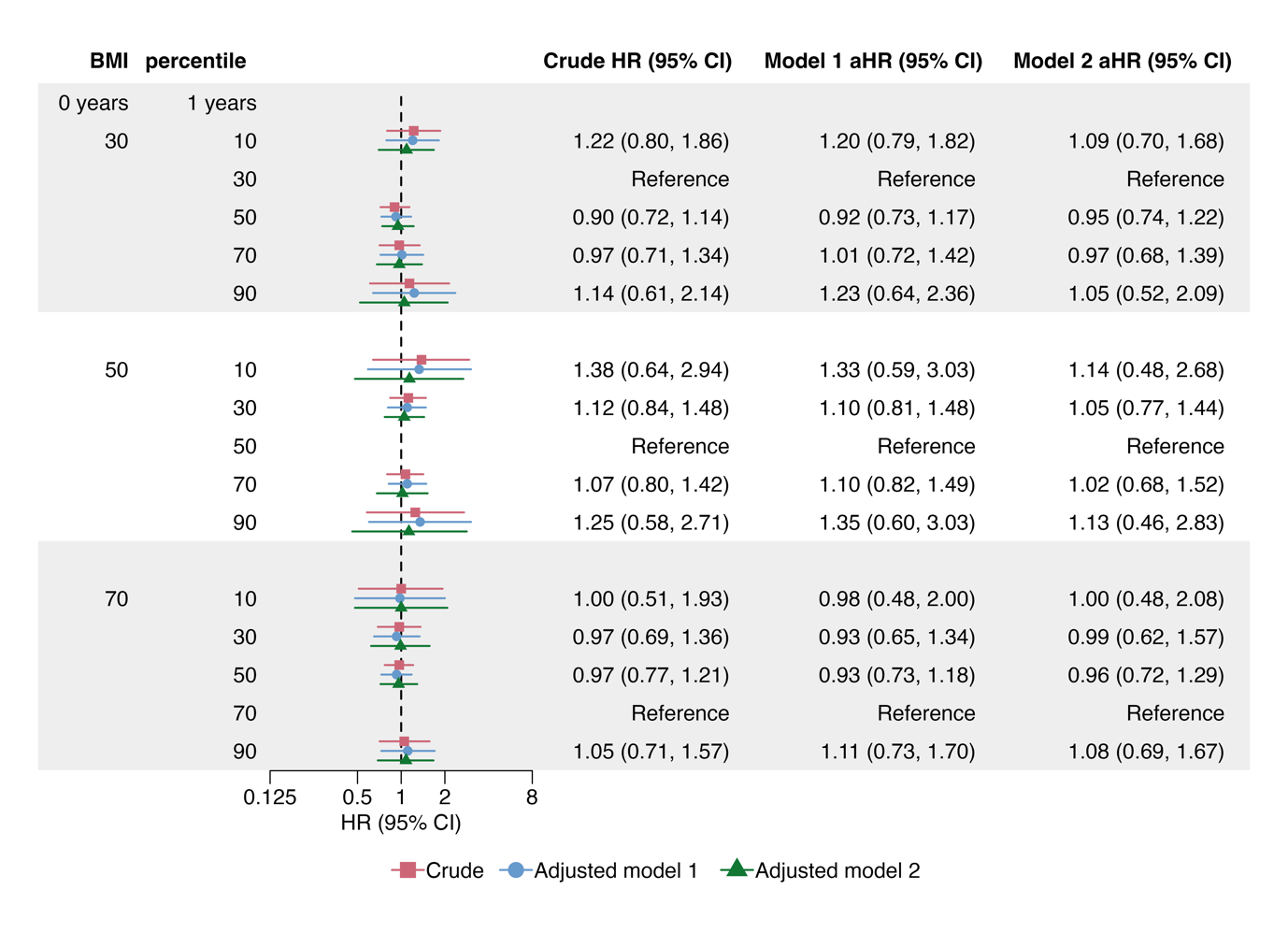


Analyses of BMI trajectories in children aged 0–1 years followed changes from the 30^th^, 50^th^, and 70^th^ BMI percentiles to the 10^th^, 30^th^, 50^th^, 70^th^, and 90^th^ percentiles, considering children who remained at the same percentile as the reference group. The number of participants in each analysis is detailed in Supplementary Figures S14 and S15.

Adjusted Model I accounted for the child’s birth weight, parental IBD and country of origin, paternal BMI status, maternal pre-pregnancy BMI, smoking and education level.

Adjusted Model II accounted for exclusive breastfeeding duration and the covariates included in Model I.

aHR: Adjusted hazard ratio, BMI: Body mass index, CI: Confidence interval, HR: Hazard ratio

# **Supplementary Figure S3.** Pooled analyses of restricted cubic spline models of the relationship between BMI percentiles trajectories at 0–1 year and ulcerative colitis.


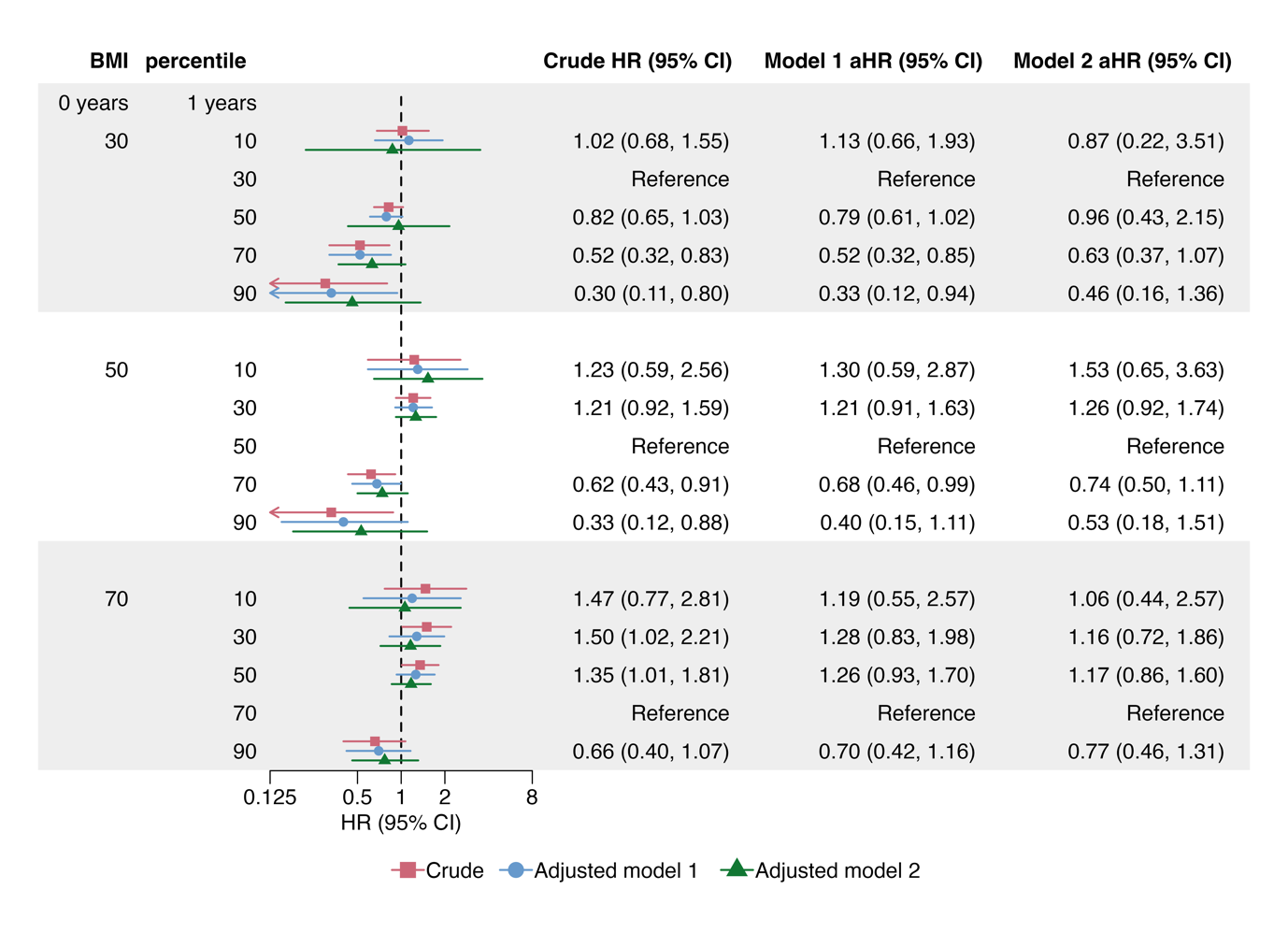


Analyses of BMI trajectories in children aged 0–1 years followed changes from the 30^th^, 50^th^, and 70^th^ BMI percentiles to the 10^th^, 30^th^, 50^th^, 70^th^, and 90^th^ percentiles, considering children who remained at the same percentile as the reference group. The number of participants in each analysis is detailed in Supplementary Figures S14 and S15.

Adjusted Model I accounted for the child’s birth weight, parental IBD and country of origin, paternal BMI status, maternal pre-pregnancy BMI, smoking and education level.

Adjusted Model II accounted for exclusive breastfeeding duration and the covariates included in Model I.

aHR: Adjusted hazard ratio, BMI: Body mass index, CI: Confidence interval, HR: Hazard ratio

# **Supplementary Figure S4.** Pooled analyses of restricted cubic spline models of the relationship between BMI percentiles trajectories at 0–3 years and inflammatory bowel disease.


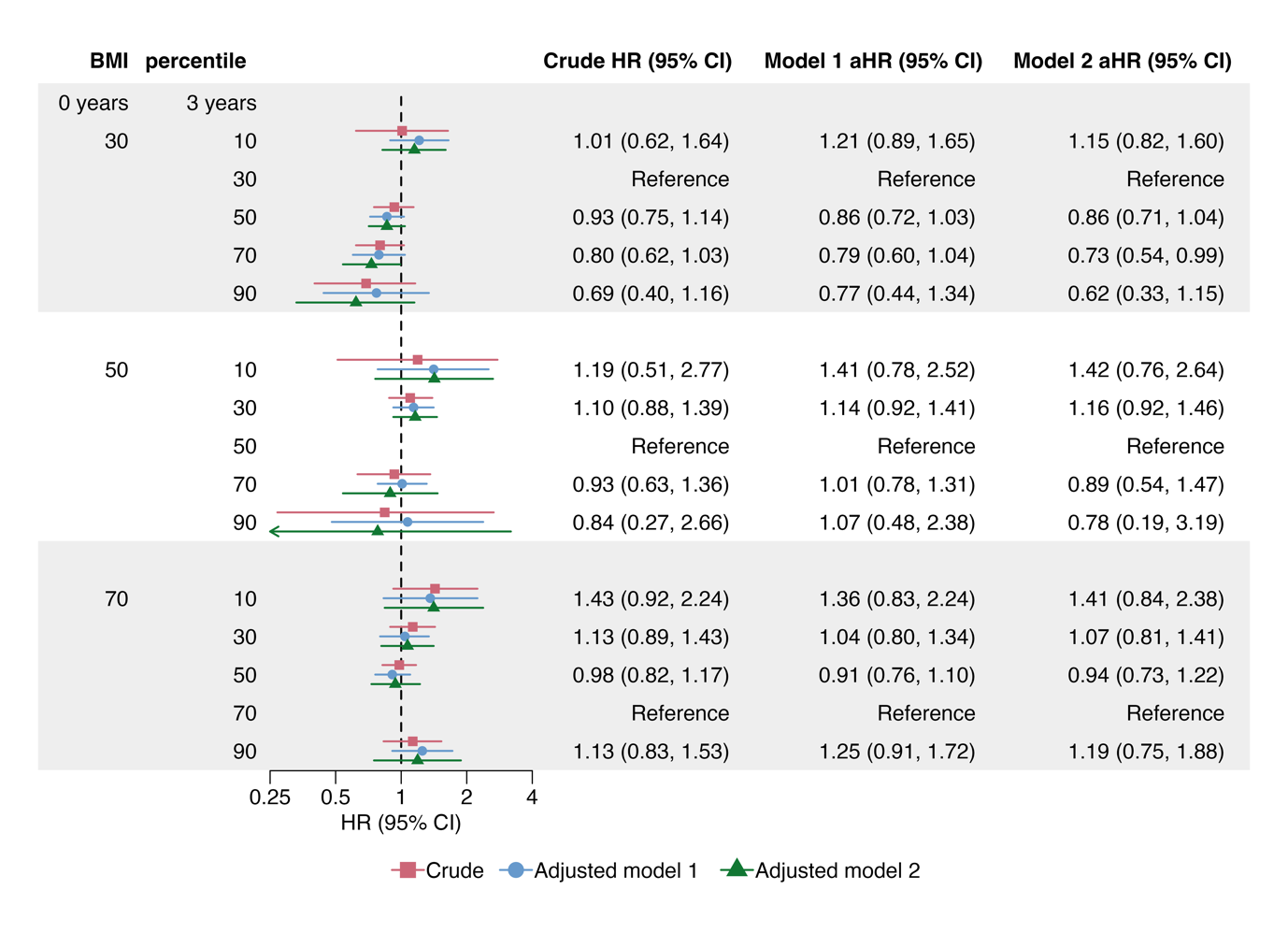


Analyses of BMI trajectories in children aged 0–3 years followed changes from the 30^th^, 50^th^, and 70^th^ BMI percentiles to the 10^th^, 30^th^, 50^th^, 70^th^, and 90^th^ percentiles, considering children who remained at the same percentile as the reference group. The number of participants in each analysis is detailed in Supplementary Figures S16–S17.

Adjusted Model I accounted for the child’s birth weight, parental IBD and country of origin, paternal BMI status, maternal pre-pregnancy BMI, smoking and education level.

Adjusted Model II accounted for exclusive breastfeeding duration and the covariates included in Model I.

aHR: Adjusted hazard ratio, BMI: Body mass index, CI: Confidence interval, HR: Hazard ratio

# **Supplementary Figure S5.** Pooled analyses of restricted cubic spline models of the relationship between BMI percentiles trajectories at 0–3 years and Crohn’s disease.


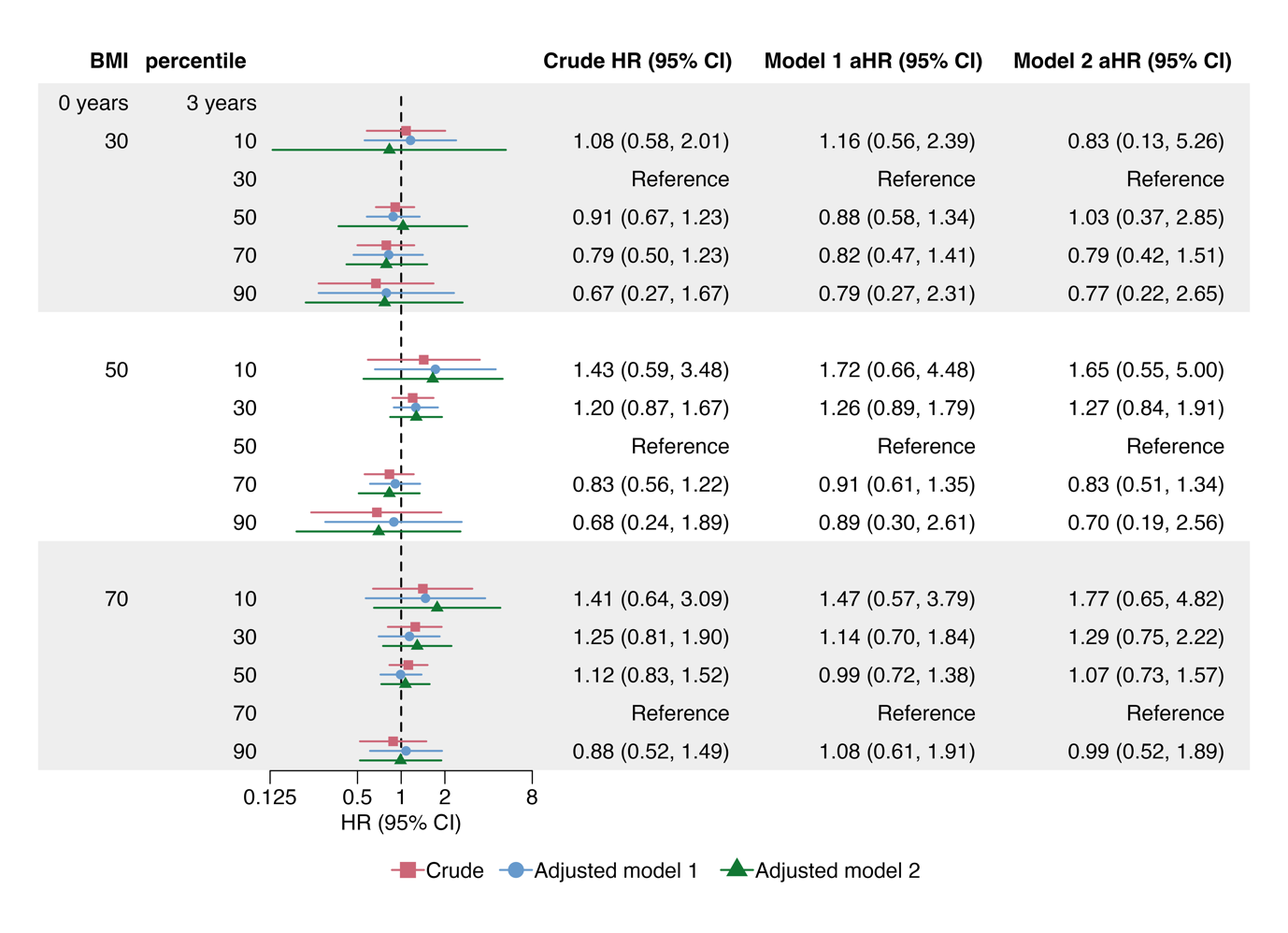


Analyses of BMI trajectories in children aged 0–3 years followed changes from the 30^th^, 50^th^, and 70^th^ BMI percentiles to the 10^th^, 30^th^, 50^th^, 70^th^, and 90^th^ percentiles, considering children who remained at the same percentile as the reference group. The number of participants in each analysis is detailed in Supplementary Figures S16–S17.

Adjusted Model I accounted for the child’s birth weight, parental IBD and country of origin, paternal BMI status, maternal pre-pregnancy BMI, smoking and education level.

Adjusted Model II accounted for exclusive breastfeeding duration and the covariates included in Model I.

aHR: Adjusted hazard ratio, BMI: Body mass index, CI: Confidence interval, HR: Hazard ratio

# **Supplementary Figure S6.** Pooled analyses of restricted cubic spline models of the relationship between BMI percentiles trajectories at 0–3 years and ulcerative colitis.


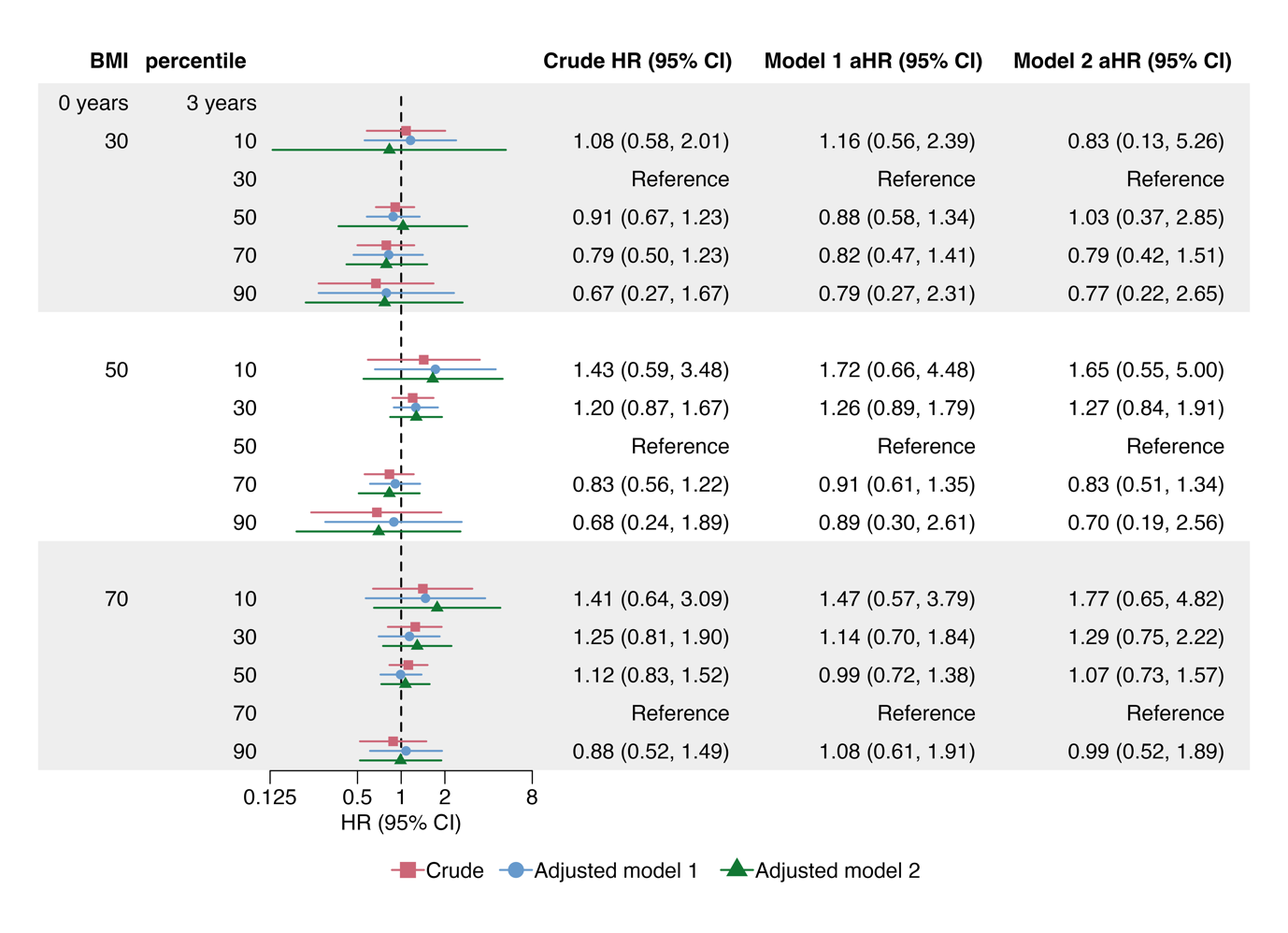


Analyses of BMI trajectories in children aged 0–3 years followed changes from the 30^th^, 50^th^, and 70^th^ BMI percentiles to the 10^th^, 30^th^, 50^th^, 70^th^, and 90^th^ percentiles, considering children who remained at the same percentile as the reference group. The number of participants in each analysis is detailed in Supplementary Figures S16–S17.

Adjusted Model I accounted for the child’s birth weight, parental IBD and country of origin, paternal BMI status, maternal pre-pregnancy BMI, smoking and education level.

Adjusted Model II accounted for exclusive breastfeeding duration and the covariates included in Model I.

aHR: Adjusted hazard ratio, BMI: Body mass index, CI: Confidence interval, HR: Hazard ratio

# **Supplementary Figure S7.** Pooled analyses of restricted cubic spline models of the relationship between BMI percentiles trajectories at 3–7/8 years and inflammatory bowel disease.


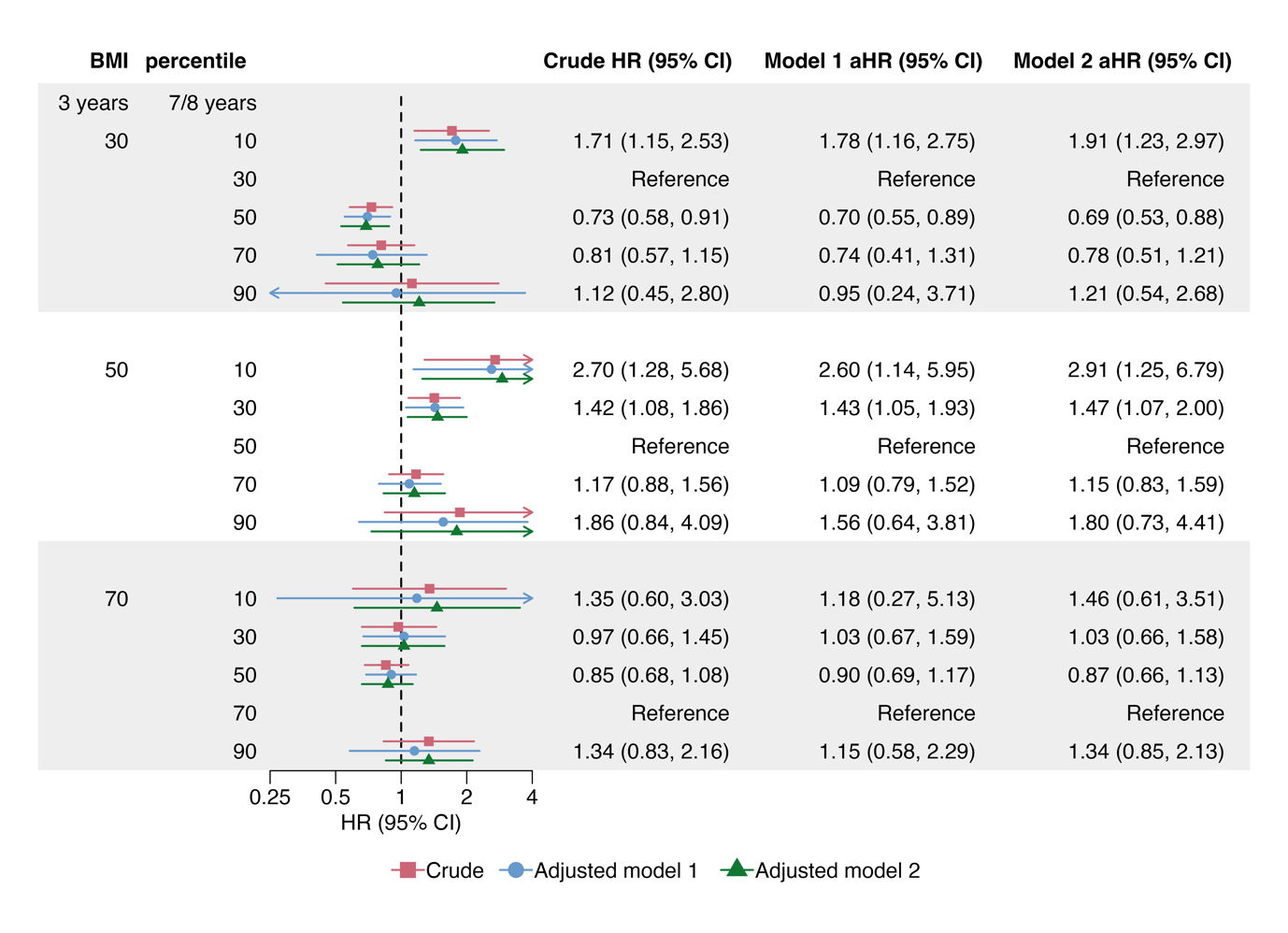


Analyses of BMI trajectories in children aged 3–7/8 years followed changes from the 30^th^, 50^th^, and 70^th^ BMI percentiles to the 10^th^, 30^th^, 50^th^, 70^th^, and 90^th^ percentiles, considering children who remained at the same percentile as the reference group. The number of participants in each analysis is detailed in Supplementary Figures S18 – S19.

Adjusted Model I accounted for the child’s birth weight, parental IBD and country of origin, paternal BMI status, maternal pre-pregnancy BMI, smoking and education level.

Adjusted Model II accounted for exclusive breastfeeding duration and the covariates included in Model I.

aHR: Adjusted hazard ratio, BMI: Body mass index, CI: Confidence interval, HR: Hazard ratio

# **Supplementary Figure S8.** Pooled analyses of restricted cubic spline models of the relationship between BMI percentiles trajectories at 3–7/8 years and ulcerative colitis.


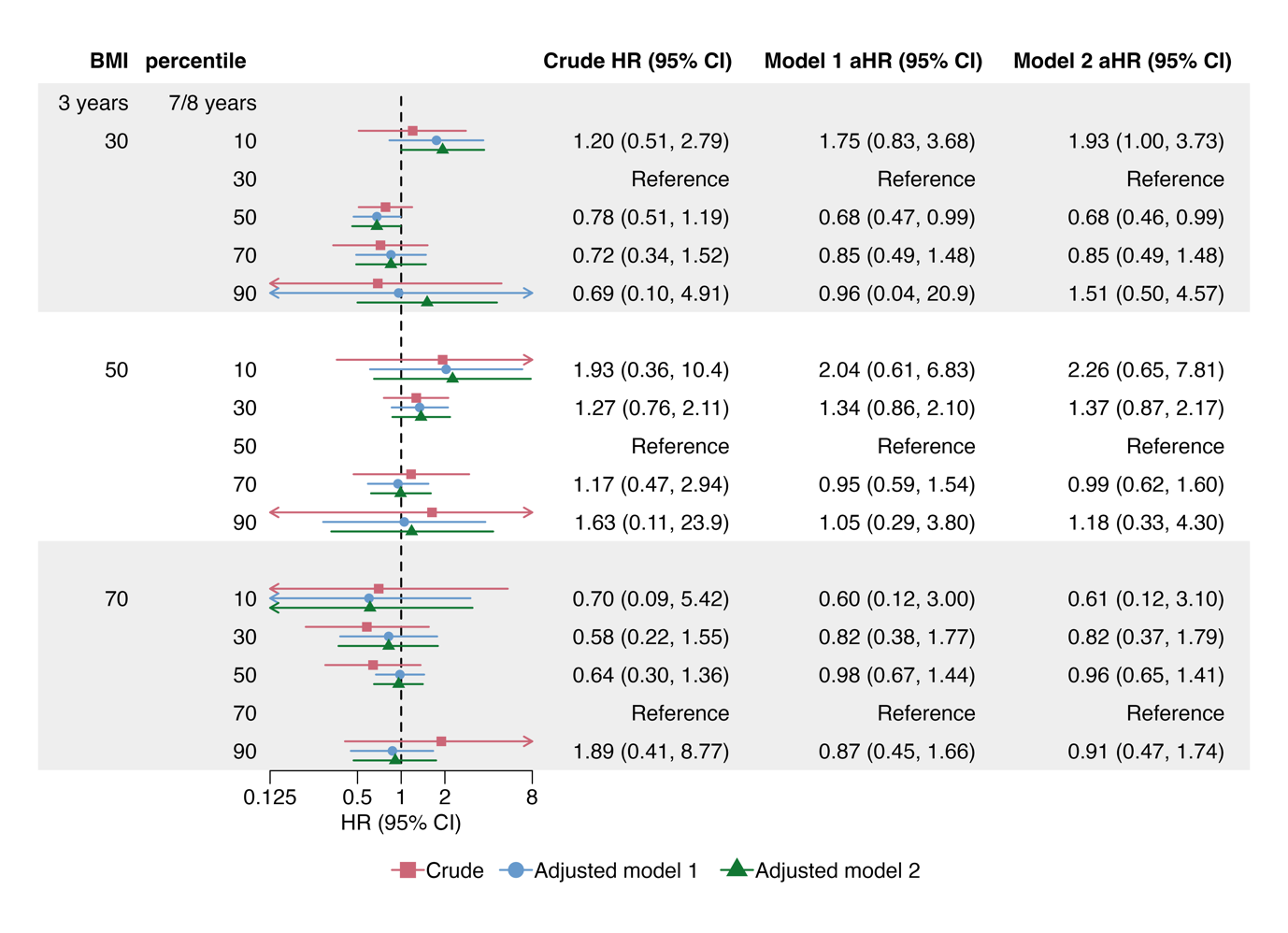


Analyses of BMI trajectories in children aged 3–7/8 years followed changes from the 30^th^, 50^th^, and 70^th^ BMI percentiles to the 10^th^, 30^th^, 50^th^, 70^th^, and 90^th^ percentiles, considering children who remained at the same percentile as the reference group. The number of participants in each analysis is detailed in Supplementary Figures S18 – S19.

Adjusted Model I accounted for the child’s birth weight, parental IBD and country of origin, paternal BMI status, maternal pre-pregnancy BMI, smoking and education level.

Adjusted Model II accounted for exclusive breastfeeding duration and the covariates included in Model I.

aHR: Adjusted hazard ratio, BMI: Body mass index, CI: Confidence interval, HR: Hazard ratio

# **Supplementary Figure S9.** Number of participants, events and incidence rates of IBD in the analyses of BMI percentile trajectories at ages 0–1 year, 0–3 years, and 3–8 years in the ABIS cohort.

Descriptive analyses of BMI trajectories in children aged 0–1 year, 0–3 years, and 3–8 years. A description of the followed BMI percentile trajectories is provided in Supplementary Figures S1–S8.

ABIS: All Babies in Southeast Sweden, BMI: Body mass index, CI: Confidence interval, N: Number

# **Supplementary Figure S9.** Number of participants, events and incidence rates of IBD in the analyses of BMI percentile trajectories at ages 0–1 year, 0–3 years, and 3–7/8 years in the MoBa cohort.

Descriptive analyses of BMI trajectories in children aged 0–1 year, 0–3 years, and 3–7/8 years. A description of the followed BMI percentile trajectories is provided in Supplementary Figures S1–S8.

BMI: Body mass index, CI: Confidence interval, MoBa: The Norwegian Mother, Father, and Child Cohort Study, N: Number

**References**

1. Mendall, M., et al., *Childhood growth and risk of inflammatory bowel disease: a population-based study of 317,030 children.* Scand J Gastroenterol, 2019. **54**(7): p. 863-868.

2. Jensen, C.B., et al., *Childhood body mass index and risk of inflammatory bowel disease in adulthood: a population-based cohort study.* Am J Gastroenterol, 2018. **113**(5): p. 694-701.

3. Mendall, M.A., et al., *Body mass index in young men and risk of inflammatory bowel disease through adult life: A population-based Danish cohort study.* Sci Rep, 2019. **9**(1): p. 6360.

4. Melinder, C., et al., *Physical Fitness in Adolescence and Subsequent Inflammatory Bowel Disease Risk.* Clin Transl Gastroenterol, 2015. **6**(11): p. e121.

5. Cole, T.J. and T. Lobstein, *Extended international (IOTF) body mass index cut-offs for thinness, overweight and obesity.* Pediatr Obes, 2012. **7**(4): p. 284-94.

6. Larsen, J.H., et al., *Higher incidence of paediatric inflammatory bowel disease by increasing latitude in Norway, but stable incidence by age.* Acta Paediatr, 2024. **113**(7): p. 1720-1727.

7. Mouratidou, N., et al., *Identification of Childhood-Onset Inflammatory Bowel Disease in Swedish Healthcare Registers: A Validation Study.* Clin Epidemiol, 2022. **14**: p. 591-600.

8. Shrestha, S., et al., *The use of ICD codes to identify IBD subtypes and phenotypes of the Montreal classification in the Swedish National Patient Register.* Scand J Gastroenterol, 2020. **55**(4): p. 430-435.

9. Forss, A., et al., *A nationwide cohort study of the incidence of inflammatory bowel disease in Sweden from 1990 to 2014.* Aliment Pharmacol Ther, 2022. **55**(6): p. 691-699.

10. Everhov, A.H., et al., *Incidence and Treatment of Patients Diagnosed With Inflammatory Bowel Diseases at 60 Years or Older in Sweden.* Gastroenterology, 2018. **154**(3): p. 518-528 e15.

11. James, P.T., et al., *The worldwide obesity epidemic.* Obes Res, 2001. **9 Suppl 4**: p. 228S-233S.

12. Guo, A., et al., *Early-life diet and risk of inflammatory bowel disease: a pooled study in two Scandinavian birth cohorts.* Gut, 2024. **73**(4): p. 590-600.

13. Lerchova, T., et al., *Physical activity in childhood and later risk of inflammatory bowel disease: A Scandinavian birth cohort study.* United European Gastroenterol J, 2023. **11**(9): p. 874-883.

14. Sigvardsson, I., et al., *Tobacco Smoke Exposure in Early Childhood and Later Risk of Inflammatory Bowel Disease: A Scandinavian Birth Cohort Study.* J Crohns Colitis, 2024. **18**(5): p. 661-670.

15. Sigvardsson, I., et al., *Childhood Socioeconomic Characteristics and Risk of Inflammatory Bowel Disease: A Scandinavian Birth Cohort Study.* Inflamm Bowel Dis, 2023.
